# Serum neurofilament light chain concentration predicts disease worsening in multiple sclerosis

**DOI:** 10.1177/13524585221097296

**Published:** 2022-06-04

**Authors:** Synne Brune, Einar A Høgestøl, Sigrid A de Rodez Benavent, Pål Berg-Hansen, Mona K Beyer, Ingvild Sørum Leikfoss, Steffan D Bos, Piotr Sowa, Cathrine Brunborg, Magi Andorra, Irene Pulido Valdeolivas, Susanna Asseyer, Alexander Brandt, Claudia Chien, Michael Scheel, Kaj Blennow, Henrik Zetterberg, Nicole Kerlero de Rosbo, Friedemann Paul, Antonio Uccelli, Pablo Villoslada, Tone Berge, Hanne F Harbo

**Affiliations:** Institute of clinical Medicine, University of Oslo, Oslo, Norway/Department of Neurology, Oslo University Hospital, University of Oslo, Oslo, Norway; Institute of Clinical Medicine, University of Oslo, Oslo, Norway/Department of Neurology, Oslo University Hospital, Oslo, Norway; Department of Psychology, University of Oslo, Oslo, Norway; Department of Ophthalmology, Oslo University Hospital, Oslo, Norway; Department of Neurology, Oslo University Hospital, Oslo, Norway; Institute of Clinical Medicine, University of Oslo, Oslo, Norway/Division of Radiology and Nuclear Medicine, Oslo University Hospital, Oslo, Norway; Department of Neurology, Oslo University Hospital, Oslo, Norway/Department of Research, Innovation and Education, Oslo University Hospital, Oslo, Norway; Institute of Clinical Medicine, University of Oslo, Oslo, Norway/Department of Neurology, Oslo University Hospital, Oslo, Norway; Division of Radiology and Nuclear Medicine, Oslo University Hospital, Oslo, Norway; Oslo Centre for Biostatistics and Epidemiology, Oslo University Hospital, Oslo, Norway; Institut d’Investigacions Biomediques August Pi Sunyer, Barcelona, Spain; Institut d’Investigacions Biomediques August Pi Sunyer, Barcelona, Spain; Experimental and Clinical Research Center, Max Delbrueck Center for Molecular Medicine and Charité-Universitaetsmedizin Berlin, Berlin, Germany; Experimental and Clinical Research Center, Max Delbrueck Center for Molecular Medicine and Charité-Universitaetsmedizin Berlin, Berlin, Germany/NeuroCure Clinical Research Center, Charité-Universitaetsmedizin Berlin, corporate member of Freie Universität Berlin, Humboldt-Universität zu Berlin, and Berlin Institute of Health, Berlin, Germany; Experimental and Clinical Research Center, Max Delbrueck Center for Molecular Medicine and Charité-Universitaetsmedizin Berlin, Berlin, Germany/NeuroCure Clinical Research Center, Charité-Universitaetsmedizin Berlin, corporate member of Freie Universität Berlin, Humboldt-Universität zu Berlin, and Berlin Institute of Health, Berlin, Germany; NeuroCure Clinical Research Center, Charité-Universitaetsmedizin Berlin, corporate member of Freie Universität Berlin, Humboldt-Universität zu Berlin, and Berlin Institute of Health, Berlin, Germany/Department of Neuroradiology, Charité-Universitaetsmedizin Berlin, corporate member of Freie Universität Berlin, Humboldt-Universität zu Berlin, and Berlin Institute of Health, Berlin, Germany; Clinical Neurochemistry Laboratory, Sahlgrenska University Hospital, Mölndal, Sweden; Clinical Neurochemistry Laboratory, Sahlgrenska University Hospital, Mölndal, Sweden/Department of Psychiatry and Neurochemistry, Institute of Neuroscience and Physiology, Sahlgrenska Academy, University of Gothenburg, Mölndal, Sweden/Department of Neurodegenerative Disease, Institute of Neurology, University College London, London, UK/UK Dementia Research Institute at UCL, London, UK/Hong Kong Center for Neurodegenerative Diseases, Shatin, Hong Kong, China; Department of Neuroscience, Rehabilitation, Ophthalmology, Genetics, Maternal and Child Health, University of Genoa, Genoa, Italy; Experimental and Clinical Research Center, Max Delbrueck Center for Molecular Medicine and Charité-Universitaetsmedizin Berlin, Berlin, Germany/NeuroCure Clinical Research Center, Charité-Universitaetsmedizin Berlin, corporate member of Freie Universität Berlin, Humboldt-Universität zu Berlin, and Berlin Institute of Health, Berlin, Germany; Department of Neuroscience, Rehabilitation, Ophthalmology, Genetics, Maternal and Child Health, University of Genoa, Genoa, Italy/Center of Excellence for Biomedical Research, University of Genoa, Genoa, Italy/IRCCS Ospedale Policlinico San Martino, Genoa, Italy; Institut d’Investigacions Biomediques August Pi Sunyer, Barcelona, Spain; Department of Research, Innovation and Education, Oslo University Hospital, Oslo, Norway/Department of Mechanical, Electronic and Chemical Engineering, Oslo Metropolitan University, Oslo, Norway; Institute of Clinical Medicine, University of Oslo, Oslo, Norway/Department of Neurology, Oslo University Hospital, Oslo, Norway

**Keywords:** Multiple sclerosis, biomarker, serum neurofilament light chain, magnetic resonance imaging, optical coherence tomography, prognosis

## Abstract

**Background:**

Serum neurofilament light (sNfL) chain is a promising biomarker reflecting neuro-axonal injury in multiple sclerosis (MS). However, the ability of sNfL to predict outcomes in real-world MS cohorts requires further validation.

**Objective:**

The aim of the study is to investigate the associations of sNfL concentration, magnetic resonance imaging (MRI) and retinal optical coherence tomography (OCT) markers with disease worsening in a longitudinal European multicentre MS cohort.

**Methods:**

MS patients (*n* = 309) were prospectively enrolled at four centres and re-examined after 2 years (*n* = 226). NfL concentration was measured by single molecule array assay in serum. The patients’ phenotypes were thoroughly characterized with clinical examination, retinal OCT and MRI brain scans. The primary outcome was disease worsening at median 2-year follow-up.

**Results:**

Patients with high sNfL concentrations (⩾8 pg/mL) at baseline had increased risk of disease worsening at median 2-year follow-up (odds ratio (95% confidence interval) = 2.8 (1.5–5.3), *p* = 0.001). We found no significant associations of MRI or OCT measures at baseline with risk of disease worsening.

**Conclusion:**

Serum NfL concentration was the only factor associated with disease worsening, indicating that sNfL is a useful biomarker in MS that might be relevant in a clinical setting.

## Introduction

Over the last two decades, neurofilament light (NfL) chain has gained increased attention as a promising biomarker in multiple sclerosis (MS). NfL is exclusively expressed in neurons and is released into the extracellular space upon axonal damage.^1^ In MS, serum NfL (sNfL) concentrations are associated with increased risk of relapses, higher neurological disability scores, increased magnetic resonance imaging (MRI) disease activity and with more severe brain and spinal cord volume loss.^2–8^ Serum NfL concentration is associated with short-term clinical outcomes,^9^ but the association with long-term clinical outcomes is less clear.^10^

Optical coherence tomography (OCT) and magnetic resonance imaging (MRI) are other markers of disease activity in MS. Reduced thickness of the ganglion cell and inner plexiform layer (GCIPL) in the retina has been reported to be associated with future disease activity in MS.^11^ Together with MRI scans of the brain and spinal cord, OCT measures can provide useful information for the prediction of long-term disability.^12^

Each of these parameters may individually provide valuable information for elucidating subsequent disease activity. However, the combination of these markers as a predictor for future disease activity in a real-world MS population has not been extensively explored. The aim of this study was to investigate the individual and the potential combined additive value of clinical, sNfL, OCT and MRI measures as markers for subsequent disease activity in a heterogeneous MS patient cohort.

## Materials and methods

### Study population

A total of 328 MS patients were included at four European MS centres (Hospital Clinic of Barcelona, Spain; Oslo University Hospital, Norway; Charité-Universitaetsmedizin Berlin, Germany; and Ospedale Policlinico San Martino, Genoa, Italy) between July 2016 and December 2017 through the ERACOSYSMED ERA-Net programme (Sys4MS project, id:43). All MS patients were invited for a 2-year follow-up visit and 280 of 328 patients were enrolled. The variability of the follow-up duration is illustrated in Supplementary eFigure 1. Complete data sets with sNfL, clinical and imaging measures were obtained from 309 and 226 MS patients at baseline and follow-up, respectively.^13^ In addition, serum samples from age- and sex-matched healthy controls (HCs) were collected at the four MS centres (59 at baseline and 30 at follow-up). For all inclusion criteria, please refer to the Supplemental Materials and Methods.

The primary study outcome was disease worsening at median 2-year follow-up, characterized by (1) ⩾3 new cerebral MRI lesions, (2) confirmed Expanded Disability Status Score (EDSS) progression, or (3) evidence of a new clinical relapse,^14^ where each component was analysed as a separate outcome parameter. EDSS progression was defined as an increase of (1) 1.5 or more if the EDSS baseline score was zero, (2) 1.0 if the baseline EDSS score was less than 5.5 and (3) 0.5 if the baseline EDSS score was ⩾5.5.^15^

The Sys4MS project followed the Declaration of Helsinki and was approved by the IRCCS Ospedale Policlinico San Martino, University of Oslo (REC ID: 2011/1846 A), Charité-Universitaetsmedizin and Hospital Clinic of Barcelona. All participants provided written informed consent prior to their inclusion in the study.

### Serum NfL chain measurements

Serum NfL concentrations were analysed using the single molecule array immunoassay (Simoa Technology; QUANTERIX, Billerica, MA, USA). See the Supplemental Materials and Methods for further details.^2^ Serum NfL percentile values were calculated from HC data (*n* = 59) and patients were grouped based on their sNfL concentrations into high (⩾8 pg/mL; ⩾75th percentile) or normal (<8 pg/mL; <75th percentile). The use of the 75th percentile cut-off was based on the distribution of MS samples in each percentile category (Supplementary eTable 1). Age-normative percentile cut-offs were calculated from a second group of HC (*n* = 309) (Blennow and Zetterberg, unpublished), dichotomizing patients based on their sNfL concentrations into high (⩾75th age-corrected percentile) or normal (<75th age-corrected percentile) (Supplementary eTable 2).

### Optical coherence tomography

Retinal OCT scans were obtained by Heidelberg Spectralis (Heidelberg Engineering, GmbH, Germany) or RS-3000 (Nidek CO., LTD., Japan), fulfilled the OSCAR-IB criteria,^16^ and were all analysed at the Berlin reading centre. See the Supplemental Materials and Methods for further details.

### Magnetic resonance imaging

MRI acquisition was harmonized across all four centres with minimum requirements for MRI scanners and sequences. Analyses of MRI data were performed at the Berlin reading centre according to a unified pipeline by experienced MRI technicians. See the Supplementary eTable 3 and the Supplemental Materials and Methods for further details.

### Statistical analysis

Statistical analyses were performed using IBM SPSS version 27 for Mac (IBM Corp., Armonk, NY, USA). Descriptive statistics are presented as either mean with standard deviation (SD), or median with range, or proportions. For comparison between groups, the *t* test or Mann–Whitney *U* test was used for continuous variables and χ^2^ or Fisher’s exact test for categorical variables. Linear regression models and partial correlation (*r_p_*) analyses were applied to test for associations between sNfL concentrations and clinical, OCT and MRI measurements at both baseline and follow-up, with sNfL and log-transformed sNfL concentrations as the outcome, adjusting for age, sex and treatment level (no treatment, active or highly active treatment). To test whether sNfL, clinical, OCT and MRI measurements could predict disease worsening, or its components, at median 2-year follow-up, univariable and multivariable logistic regression analyses were applied including sNfL concentration ⩾ 8 pg/mL or above the age-corrected 75th percentile, GCIPL thickness, pRNFL thickness, T2 lesion volume, normalized brain volumes, average time used at Nine-Hole Peg Test (9-HPT), age, and treatment level as covariates in the logistic regression analyses. For the sensitivity analyses, different sNfL percentile cut-offs were used to examine whether the probability of disease worsening increased with each category of higher sNfL-level percentile. Before performing the multivariable regression analysis, possible multicollinearity of the covariates was explored using a correlation coefficient ⩾0.7 as a limit for multicollinearity. All tests were two-sided and a 5% significance level was used. The *p* values were not adjusted for multiple testing. OCT measures were missing in 27% of the patients, and to investigate the possible uncertainty due to missing data in the multivariable logistic regression models with OCT measures, sensitivity analysis of imputation was performed as described in the Supplemental Materials and Methods.

## Results

### Baseline characteristics

Demographic and clinical characteristics of the study population are shown in Tables 1–3 and in Supplementary eTable 4. The sNfL concentrations by age are separately presented as scatter plots for HCs and MS patients in Supplementary eFigure 2. Serum NfL concentrations at baseline were significantly higher in patients with progressive MS (PMS) compared with HCs, adjusted for age and sex (Table 2). When subgrouping the patients based on treatment level, our analyses showed that untreated PMS patients had significantly higher sNfL concentrations than the HC group (Supplementary eTable 5 and Supplementary eFigure 3).

**Table 1.** Demographic and clinical features of the study population.

	Baseline		2-year follow-up	
	RRMS (*n* = 257)	PMS (*n* = 52)	RRMS (*n* = 188)	PMS (*n* = 38)
Age, years, mean (SD)	40.9 (±8.6)	54.1 (± 7.4)	42.6 (±8.7)	56.4 (±7.4)
Sex, *n* (%)				
Female	186 (72.4)	31 (59.6)	137 (73.0)	23 (60.5)
MS classification, *n* (%)				
CIS	4 (1.6)		3 (1.6)	
RRMS	253 (98.4)		185 (98.4)	
SPMS		24 (46.2)		19 (50.0)
PPMS		28 (53.8)		19 (50.0)
Age at MS onset, mean, years (SD)	30.7 (±8.4)	35.3 (±11.1)		
Disease duration, median, years (range)	8.4 (0.5–34.3)	17.4 (1.4–43.5)		
Follow-up time, median, years (range)			1.9 (0.7–3.4)	2.1 (1.0–2.4)
Follow-up time, mean, years (SD)			1.9 (±0.4)	1.9 (±0.3)
Disease-modifying treatment				
None, *n* (%)	56 (22.0)	34 (65.0)	32 (18.0)	19 (51.5)
Active treatment, *n* (%)	130 (50)	7 (14)	81 (45)	3 (8)
Interferon	37 (14.4)	3 (5.8)	13 (7.2)	1 (2.7)
Glatiramer acetate	33 (12.8)	3 (5.8)	19 (10.5)	1 (2.7)
Teriflunomid	27 (10.5)	0 (0)	19 (10.5)	0 (0)
Fumarate	33 (12.8)	1 (1.9)	30 (16.6)	1 (2.7)
Highly active treatment, *n* (%)	71 (28.0)	11 (21.0)	68 (37.0)	15 (40.5)
Fingolimod	35 (13.6)	2 (3.8)	27 (14.9)	2 (5.4)
Natalizumab	25 (9.7)	1 (1.9)	19 (10.5)	1 (2.7)
Alemtuzumab	8 (3.1)	1 (1.9)	12 (6.6)	0 (0)
Rituximab	1 (0.4)	6 (11.5)	4 (2.2)	4 (10.8)
Ocrelizumab	0 (0)	1 (1.9)	4 (2.2)	8 (21.6)
Daclizumab	2 (0.8)	0 (0)	0 (0)	0 (0)
Cladribine	0 (0)	0 (0)	2 (1.1)	0 (0)

RRMS: relapsing-remitting MS; PMS: progressive MS; MS: multiple sclerosis; SD: standard deviation; CIS: clinically isolated syndrome; SPMS: secondary progressive MS; PPMS: primary progressive MS.

Descriptive statistics are presented as either mean with standard deviation (SD), or median with range or proportions.

**Table 2.** Clinical, MRI and OCT measures in the study population.

	Baseline						2-year follow-up					
	HC (*n* = 59)	RRMS (*n* = 257)	PMS (*n* = 52)	*p* ^a^	*p* ^b^	*p* ^c^	HC (*n* = 30)	RRMS (*n* = 188)	PMS (*n* = 38)	*p* ^a^	*p* ^b^	*p* ^c^
sNfL (pg/mL), median (range)	6.0 (2.2–21.6)	6.7 (2.2–93.2)	10.7 (4.2–28.4)	0.12	0.295	**0.03**	7.1 (3.0–16.2)	6.6 (2.3–45.5)	11.0 (4.3–32.8)	0.101	0.954	0.207
EDSS, median (range)		2.0 (0–6.5)	5.0 (2.0–8.0)	**<0.001**				1.5 (0–7.0)	6.0 (2–8.0)	**<0.001**		
9-HPT, median (range)		19.6 (14.4–48.3)	25.7 (17.7–49.5)	**<0.001**				19.7 (14.8–45.3)	26.4 (17.6–59.6)	**<0.001**		
25FWT, median (range)		4.3 (2.5–71.0)	12.1 (4.4–44.0)	**<0.001**				4.2 (2.3–22.2)	9.4 (5.5–59.8)	**<0.001**		
SDMT, median (range)		55.0 (20–102)	41 (14–69)	**0.015**				53.0 (25–110)	43.5 (23–75)	0.148		
Brain T2 Lesion count, median (range)		25 (1–204)	34.5 (4–232)	**0.005**				27 (0–197)	35 (5–103)	0.4		
Brain T2 Lesion volume, median (range)		3.5 (0.1–64.5)	15.0 (0.5–54.1)	**<0.001**				4.0 (0.1–57.0)	15.2 (0.9–54.4)	**<0.001**		
Normalized brain volume, mean (SD)		1519.4 (±83.1)	1437.9 (±98.3)	0.099				1460.0 (±65.0)	1411.7 (±79.6)	0.105		
Normalized grey matter volume, mean (SD)		799.82 (±60.95)	740.1 (±59.86)	0.218				785.9 (±45.5)	748.8 (±49.6)	0.645		
Normalized white matter volume, mean (SD)		719.8 (±68.4)	697.8 (±80.0)	0.368				674.0 (±42.0)	663.0 (±53.0)	**0.049**		
Thalamus volume, mean (SD)		15.16 (±1.79)	13.4 (±2.2)	**<0.001**				14.9 (±2.4)	13.4 (±2.6)	0.129		
pRNFL non-ON (μm), median (range)		103.3 (77.0–138.9) *n* = 195	104.9 (76.8–126.7) *n* = 37	0.98				104.0 (77.4–151.9) *n* = 150	105.5 (82.2–123.5) *n* = 23	0.748		
GCIPL non-ON (μm), median (range)		67.4 (47.8–85.6) *n* = 207	67.5 (53.7–78.3) *n* = 36	**0.047**				66.3 (48.5–83.4) *n* = 140	66.5 (45.5–80.5) *n* = 24	0.313		

MRI: magnetic resonance imaging; OCT: optical coherence tomography; HC: healthy control; RRMS: relapsing remitting MS; PMS: progressive multiple sclerosis; sNfL: serum neurofilament light chain; EDSS: Expanded Disability Status Score; 9-HPT: 9HolePegTest; 25FWT: 25 foot walk test; SDMT: symbol digit modalities test; SD: standard deviation; pRNFL non-ON: peripapillary retinal nerve fiber layer thickness in nonoptic neuritis eye; GCIPL non-ON: ganglion cell and inner plexiform layer in nonoptic neuritis eye.Descriptive statistics are presented as either mean with standard deviation (SD), or median with range. The p values were derived using linear regression models adjusted for age, sex and treatment level. In bold are shown significant p values. The p values were not adjusted for multiple testing.

a p = p value for the RRMS vs PMS comparison.

b p = p value for the HC vs RRMS comparison.

c p = p value for the HC vs PMS comparison.

**Table 3.** Demographic and clinical features of patients with disease worsening.

	Patients with disease worsening (*n* = 87)	Patients without disease worsening (*n* = 109)
Age, years, mean (SD)	42.9 (±9.8)	41.9 (±9.7)
Sex, *n* (%)		
Female	55 (63.2)	86 (78.9)
MS classification, *n* (%)		
CIS	2 (2.3)	2 (1.8)
RRMS	64 (73.6)	97 (89.0)
SPMS	10 (11.5)	8 (7.3)
PPMS	11 (12.6)	2 (1.8)
Age MS onset, years, mean (SD)	31.4 (±9.1)	30.5 (±8.5)
Disease duration, years, median (range)	8.9 (0.5–43.5)	8.6 (0.5–34.7)
Follow-up time, years, median (range)	2.0 (0.95–3.38)	1.9 (0.7–2.72)
Disease-modifying treatment		
None, *n* (%)	29 (33.3)	22 (20.2)
Active treatment, *n* (%)	36 (41.4)	50 (45.9)
Interferon	12 (33.3)	11 (22.0)
Glatiramer acetate	10 (27.8)	8 (16.0)
Teriflunomid	6 (16.7)	14 (28.0)
Fumarate	8 (22.2)	17 (34.0)
Highly active treatment, *n* (%)	22 (25.3)	37 (33.9)
Fingolimod	10 (45.5)	16 (43.2)
Natalizumab	7 (31.8)	13 (35.1)
Alemtuzumab	2 (9.1)	5 (13.5)
Rituximab	2 (9.1)	2 (5.4)
Ocrelizumab	1 (4.5)	0 (0)
Daclizumab	0 (0)	1 (2.7)
sNfL (pg/mL) median (range)	8.4 (2.8–93.2)	6.7 (2.7–33.3)
sNfL ⩾ 75th percentile^a^, *n* (%)	49 (56.3)	46 (42.2)
sNfL ⩾ 8 pg/mL, *n* (%)	49 (56.3)	36 (33.0)
⩾3 new cerebral MRI lesions, *n* (%)	34 (39.1)	0 (0)
EDSS progression, *n* (%)	39 (44.8)	0 (0)
Relapse, *n* (%)	33 (37.9)	0 (0)

SD: standard deviation; CIS: clinically isolated syndrome; RRMS: relapsing-remitting MS; SPMS: secondary progressive MS; PPMS: primary progressive MS; MRI: magnetic resonance imaging; EDSS: Expanded Disability Status Score; MS: multiple sclerosis.

Disease worsening = ⩾3 new cerebral MRI lesions and/or confirmed EDSS progression and/or evidence of a new clinical relapse. Descriptive statistics are presented as either mean with standard deviation (SD), or median with range or proportions.

a Cut-off 75th percentile: 20–29 years ⩾ 4.325 pg/mL, 30–34 years ⩾ 6.60 pg/mL, 35–39 years ⩾ 7.05 pg/mL, 40–44 years ⩾ 6.825 pg/mL, 45–49 years ⩾ 7.075 pg/mL, 50–54 years ⩾ 8.225 pg/mL, 55–59 years ⩾ 11.15 pg/mL, 60–69 years ⩾ 12.95 pg/mL.

### Association between sNfL and clinical, MRI and OCT measures at baseline and follow-up

**Figure 1.**
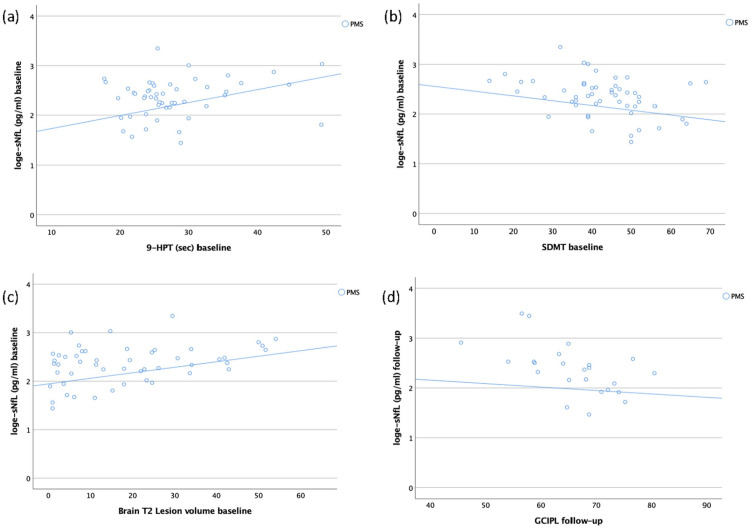
Scatter plots depicting the association (partial correlations) between log-transformed sNfL concentrations and clinical, radiological and OCT parameters at baseline or follow-up. (a) sNfL concentration at baseline and 9-HPT at baseline in patients with PMS (*r_p_*/*p* = 0.38/0.01). (b) sNfL concentration at baseline and SDMT at baseline in patients with PMS (*r_p_*/*p* = –0.32/0.03). (c) sNfL concentration at baseline and brain T2 lesion volume at baseline in patients with PMS (*r_p_*/*p* = 0.39/0.01). (d) sNfL concentration at follow-up and GCIPL at follow-up in patients with PMS (*r_p_*/*p* = –0.52/0.02). sNfL: serum neurofilament light chain; RRMS: relapsing remitting multiple sclerosis; PMS: progressive multiple sclerosis; 9-HPT: 9HolePegTest; SDMT: symbol digit modalities test; GCIPL: ganglion cell and inner plexiform layer; *r_p_*: partial correlation coefficient (corrected for age, sex and treatment level). The *p* values were not adjusted for multiple testing.

Correlation analyses of log-transformed sNfL concentrations with clinical, MRI and OCT measures at baseline and follow-up are presented in Figure 1 and in Supplementary eTable 6. In the PMS group, higher sNfL concentrations at baseline were significantly associated with slower performance on 9-HPT (*r_p_* = 0.38, *p* = 0.01), lower scores on the symbol digit modalities test (SDMT) (*r_p_* = –0.32, *p* = 0.03), higher T2 lesion count (*r_p_* = 0.41, *p* = 0.004), and increased T2 lesion volume (*r_p_* = 0.39, *p* = 0.01) at baseline. Furthermore, higher sNfL concentrations at follow-up were significantly associated with higher T2 lesion count (*r_p_* = 0.36, *p* = 0.04) and reduced thickness of GCIPL (*r_p_* = –0.52, *p* = 0.02) at follow-up among the PMS patients.

In the relapsing-remitting MS (RRMS) group, higher baseline sNfL concentrations were significantly associated with higher T2 lesion count at baseline (*r_p_* = 0.15, *p* = 0.02). In addition, in this patient group higher sNfL concentrations at follow-up were significantly associated with slower performance on both 9-HPT (*r_p_* = 0.24, *p* = 0.003) and 25-foot walk test (T25FWT) (*r_p_* = 0.31, *p* < 0.001) at follow-up. The presence of new lesions (*r_p_* = 0.28, *p* < 0.001) and increasing lesion volumes (*r_p_* = 0.21, *p* = 0.01) at follow-up significantly correlated with higher concentrations of sNfL at baseline in the RRMS group.

### Association of sNfL, clinical, MRI and OCT measures with disease worsening

**Figure 2.**
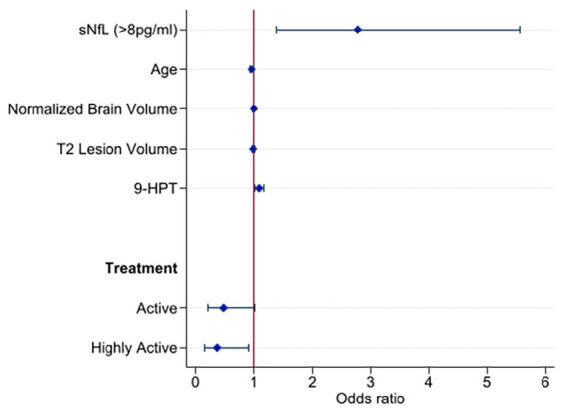
A forest plot depicting the multivariable logistic regression model 2 for disease progression at 2-year follow-up. No treatment (odds ratio = 1.0) is used as a reference category for treatment. sNfL: serum neurofilament light chain; 9-HPT: 9HolePegTest.

We then investigated whether high sNfL concentrations at baseline, thinner pRNFL, thinner GCIPL, higher T2 lesion volume, lower normalized total brain volume, slower performance on 9-HPT or DMT category separately were associated with disease worsening at median 2-year follow-up. Patients with high sNfL concentrations (⩾8 pg/mL) at baseline had higher risk of disease worsening at follow-up (odds ratio (OR) (95% confidence interval (CI)) = 2.8 (1.5–5.3), *p* = 0.001) (Figure 2 and in Supplementary eTable 7). The risk of disease worsening was also significantly increased in the univariable model using the 80th percentile (OR (95% CI) = 1.98 (1.02–3.8), *p* = 0.043) (Supplementary eTable 8). When analysing the association of sNfL concentrations with single components of disease worsening, patients with high sNfL (⩾8 pg/mL) at baseline had increased risk of developing new T2 lesions (OR (95% CI) = 3.97 (1.7–9.3), *p* = 0.002) and of experiencing a new clinical relapse (OR (95% CI) = 3.3 (1.38–7.8), *p* = 0.007) in the follow-up period, but not of EDSS progression (Table 4). The risk of disease worsening (OR (95% CI) = 1.07 (1.01–1.14), *p* = 0.027) and of experiencing a new clinical relapse in the follow-up period (OR (95% CI) = 1.07 (1.01–1.13), *p* = 0.033) was also significantly increased in the univariable models using sNfL as a continuous variable (Table 4). In addition, slower performance on 9-HPT was significantly associated with disease worsening at follow-up (OR (95% CI) = 1.09 (1.02–1.17), *p* = 0.009). Neither thinner GCIPL nor pRNFL, increased T2 lesion volume nor lower normalized total brain volume showed associations with disease worsening at follow-up. The data indicated that patients with active or highly active treatment had less disease worsening compared with untreated patients.

**Table 4.** Univariable models for disease worsening, or its components individually, age adjusted.

	Disease worsening (*n* = 196)			⩾3 new cerebral MRI lesions (*n* = 205)			EDSS progression (*n* = 206)			Relapse (*n* = 177)		
	OR	95% CI	*p* value	OR	95% CI	*p* value	OR	95% CI	*p* value	OR	95% CI	*p* value
sNfL ⩾ 75th (NfL ⩾ 8.0 pg/mL)	**2.8**	**1.49–5.31**	**0.001**	**3.97**	**1.7–9.3**	**0.002**	1.84	0.85–3.96	0.121	**3.3**	**1.38–7.81**	**0.007**
sNfL ⩾ 75th^a^	**1.8**	**1.00–3.12**	**0.050**	**2.3**	**1.1–4.9**	**0.034**	1.201	0.6–2.4	0.61	1.978	0.914–4.281	0.083
sNfL (pg/mL, continuous)	**1.07**	**1.01–1.14**	**0.027**	1.05	0.99–1.09	0.069	1.01	0.97–1.05	0.57	**1.07**	**1.01–1.13**	**0.033**
Age^b^	1.01	0.98–1.04	0.455	0.99	0.95–1.02	0.417	1.03	0.99–1.07	0.07	0.98	0.95–1.02	0.43
Brain T2 Lesion volume	1.01 (*n* = 195)	0.98–1.04	0.59	1.01	0.97–1.04	0.803	1.00 (*n* = 205)	0.97–1.04	0.79	1.00 (*n* = 176)	0.96–1.04	0.93
Normalized brain volume	1.00 (*n* = 194)	0.99–1.00	0.89	**1.01** (*n* = **204**)	**1.00–1.01**	**0.033**	0.99 (*n* = 204)	0.99–1.003	0.37	1.00 (*n* = 175)	0.99–1.01	0.69
GCIPL non-ON, continuous	1.03 (*n* = 154)	0.99–1.07	0.161	1.03 (*n* = 161)	0.98–1.09	0.23	1.02 (*n* = 161)	0.97–1.08	0.36	0.99 (*n* = 140)	0.94–1.05	0.911
pRNFL non-ON, continuous	0.99 (*n* = 147)	0.97–1.02	0.67	1.01 (*n* = 155)	0.98–1.04	0.63	0.99 (*n* = 155)	0.95–1.02	0.43	0.98 (*n* = 134)	0.94–1.02	0.26
9-HPT (per 1 second)	**1.09** (*n* = **190**)	**1.02–1.17**	**0.009**	1.05 (*n* = 198)	0.98–1.12	0.15	1.03 (*n* = 199)	0.97–1.09	0.28	**1.12** (*n* = **174**)	**1.03–1.2**	**0.005**
No treatment	1			**1**			1					
Active treatment	0.55 (*n* = 196)	0.27–1.13	0.103	1.03 (*n* = 205)	0.42–2.5	0.95	0.51 (*n* = 206)	0.21–1.22	0.13	1.02 (*n* = 177)	0.42–2.48	0.97
Highly active treatment	0.46 (*n* = 196)	0.21–1.0	0.056	0.52 (*n* = 205)	0.18–1.16	0.25	1.12 (*n* = 206)	0.45–2.8	0.80	**0.24** (*n* = **177**)	**0.07–0.88**	**0.031**

MRI: magnetic resonance imaging; EDSS: Expanded Disability Status Score; OR: odds ratio; CI: confidence interval; sNfL: serum neurofilament light chain; GCIPL non-ON: ganglion cell and inner plexiform layer in nonoptic neuritis eye; pRNFL non-ON: peripapillary retinal nervefiber layer thickness in nonoptic neuritis eye; 9-HPT: 9HolePegTest.

Disease worsening = ⩾3 new cerebral MRI lesions and/or confirmed EDSS progression and/or evidence of a new clinical relapse. Results are presented with OR, 95% CI and *p* value. Significant *p* values as well as the corresponding OR and CI are shown in bold. The *p* values were not adjusted for multiple testing.

a Cut-off 75th percentile: 20–29 years ⩾ 4.325 pg/mL, 30–34 years ⩾ 6.60 pg/mL, 35–39 years ⩾ 7.05 pg/mL, 40–44 years ⩾ 6.825 pg/mL, 45–49 years ⩾ 7.075 pg/mL, 50–54 years ⩾ 8.225 pg/mL, 55–59 years ⩾ 11.15 pg/mL, 60–69 years ⩾ 12.95 pg/mL.

b Not age adjusted.

Since age is the single most important factor impacting sNfL concentrations in HCs,^9^ we extended our analyses using age-normative cut-offs. Patients with high sNfL concentrations (⩾75th age-corrected percentile) at baseline had higher risk of disease worsening at follow-up (OR (95% CI) = 1.8 (1.0–3.12), *p* = 0.050) (Supplementary eTable 9). The risk of disease worsening was also significantly increased in the univariable models using sNfL concentrations ⩾80th age-corrected percentile (OR (95% CI) = 2.1 (1.2–3.8), *p* = 0.009), ⩾85th age-corrected percentile (OR (95% CI) = 2.3 (1.3–4.1), *p* = 0.005) and ⩾90th age-corrected percentile (OR (95% CI) = 2.4 (1.3–4.3), *p* = 0.005) as cut-offs (Supplementary eTable 10). Patients with high sNfL (⩾75th age-corrected percentile) at baseline had increased risk of developing new T2 lesions (OR (95% CI) = 2.3 (1.1–4.9), *p* = 0.034), but not of experiencing a new clinical relapse or of EDSS progression in the follow-up period. In addition, patients with sNfL ⩾80th, 85th and 90th age-corrected percentiles at baseline had increased risk of developing new T2 lesions, and patients with sNfL ⩾80th and the 85th age-corrected percentiles had increased risk of experiencing a new clinical relapse in the follow-up period.

To analyse whether sNfL, MRI and OCT parameters combined showed stronger associations with disease worsening than each parameter alone, we combined sNfL, MRI and OCT measures in multivariable models. See the Supplemental Results and Supplementary eTables 7 and 9 for further details.

## Discussion

This multicentre, real-life study revealed increased risk of disease worsening after median 2 years in patients with elevated sNfL concentrations at baseline. The association between sNfL concentration and disease worsening was first explored using a pre-specified cut-off (⩾8 pg/mL). These analyses revealed a 2.8-fold increased risk of disease worsening, a 4.0-fold increased risk of new T2 lesions and a 3.3-fold increased risk of relapse activity after 2 years. There was a trend, although not statistically significant, for a positive association between high sNfL at baseline and the risk of EDSS progression at follow-up. Defining age-normative cut-offs in this analysis was not possible due to limited number of HCs (*n* = 59) that were analysed at the same time as the patient samples. We then explored the association between sNfL concentration and disease worsening using age-normative cut-offs (⩾75th percentile) based on a second group of HCs (*n* = 309). This analysis revealed a 1.8-fold increased risk of disease worsening, gradually increasing using a higher percentile as cut-off. The risk of new MRI lesions and a new relapse in the follow-up period was also evident in these analyses using age-normative cut-offs. Finally, the risk of disease worsening, and of experiencing a new clinical relapse in the follow-up period, was also significantly increased in the analysis using sNfL as a continuous variable. Our study showed no independent association between MRI or OCT measures and disease worsening. Overall, sNfL was the only factor associated with disease worsening in this patient population.

Disanto et al.^2^ have previously reported that patients with sNfL concentrations above the 80th HCs-based percentiles at baseline had higher risk of relapses and EDSS worsening after 3 years, supporting our findings. This was confirmed in another cohort,^8^ where the probability of EDSS worsening gradually increased with higher sNfL percentile categories. A recent study by Kuhle et al.^17^ demonstrated that the combination of sNfL concentration and brain atrophy provided a more robust prediction for long-term disease progression than sNfL concentrations alone, whereas Zimmermann et al. reported that the presence of both high sNfL and thin GCIPL was a stronger risk factor for future disease activity than each parameter individually.^18^ We found no additive effect of sNfL, MRI and OCT measures as risk factors for disease worsening. Of note, the patients followed by Kuhle et al. were not using highly active treatment and their outcome measure was long-term disease progression measured after 8 or 15 years. In contrast, our patient population was mainly treated with active or highly active DMTs and had low disability scores, and disease worsening was measured only after 2 years. The observed association between disease worsening and high sNfL concentration, but not with MRI measures in our patient population, is possibly due to high sensitivity for sNfL as a biomarker of ongoing neuro-axonal degeneration even in early stages of the disease,^19^ as opposed to cerebral atrophy, which advances more slowly over time and is more pronounced in later stages of the disease course.^12,20,21^

Our cross-sectional analyses confirm previous reports stating that sNfL concentrations are associated with clinical disability and cognitive performance, the number of T2 lesions, T2 lesion volume and the presence of new T2 lesions.^2,5,22,23^ However, we found no significant associations between sNfL and normalized brain volume measures, in contrast to what was observed by Barro et al.^8^ One explanation for this could be that our patient population had lower disability scores at baseline and were treated with more highly effective DMTs. In addition, the follow-up time was relatively short.

Previous studies have demonstrated higher sNfL concentrations in MS patients in general compared with HCs, but we found no significant differences in sNfL concentrations between RRMS patients and HCs in our cohort. This could also be because we have included well-treated patients where DMT reduces neuro-axonal damage and thereby lowers sNfL release. In contrast, we found that untreated PMS patients had significantly higher sNfL concentrations compared with HCs. Interestingly, PMS patients on active or highly active treatment had comparable sNfL concentrations with HCs, suggesting that DMT might also influence the pathological processes in PMS patients. These results need to be interpreted with caution because of the low number of PMS patients on active or highly active treatment. However, this hypothesis is supported by other studies showing that NfL concentrations both in serum and in cerebrospinal fluid (CSF) are reduced after initiating DMT in PMS.^24,25^

Our study did not identify significant associations between sNfL and GCIPL or pRNFL at baseline, but we found a significant correlation between sNfL and GCIPL at follow-up among the PMS patients. OCT studies in MS show that GCIPL parameters are often affected early in the disease course even in patients without previous optic neuritis.^26^ This is interpreted as a sign of the ongoing neurodegenerative process. When the disease activity develops further, the pRNFL may also be affected. Increased sNfL levels reflecting neuro-axonal retinal damage might be more pronounced in later and more progressive stages of the disease and may not be evident among RRMS patients using efficient DMT.^27^ This could explain why there was only an association between sNfL and GCIPL in PMS patients in our cohort and not among stable RRMS patients.

A strength of this study is the large MS patient population recruited through a prospective longitudinal European MS-specialized multicentre study. To our knowledge, this is the first study to investigate the associations of sNfL, clinical, MRI and OCT measures with disease worsening in a real-world MS patient population. One limitation of the study is the short follow-up of our cohort. Our results are indicative of the short-term prognosis, but may not be informative for the long-term prognosis of MS.

## Conclusion

This study showed that high sNfL concentration was associated with disease worsening in a real-world MS population. We conclude that sNfL is a promising biomarker in MS that might be relevant in a clinical setting.

## Supplemental Material

sj-docx-10-msj-10.1177_13524585221097296 – Supplemental material for Serum neurofilament light chain concentration predicts disease worsening in multiple sclerosis Click here for additional data file. Supplemental material, sj-docx-10-msj-10.1177_13524585221097296 for Serum neurofilament light chain concentration predicts disease worsening in multiple sclerosis by Synne Brune, Einar A Høgestøl, Sigrid A de Rodez Benavent, Pål Berg-Hansen, Mona K Beyer, Ingvild Sørum Leikfoss, Steffan D Bos, Piotr Sowa, Cathrine Brunborg, Magi Andorra, Irene Pulido Valdeolivas, Susanna Asseyer, Alexander Brandt, Claudia Chien, Michael Scheel, Kaj Blennow, Henrik Zetterberg, Nicole Kerlero de Rosbo, Friedemann Paul, Antonio Uccelli, Pablo Villoslada, Tone Berge and Hanne F Harbo in Multiple Sclerosis Journal

sj-docx-11-msj-10.1177_13524585221097296 – Supplemental material for Serum neurofilament light chain concentration predicts disease worsening in multiple sclerosis Click here for additional data file. Supplemental material, sj-docx-11-msj-10.1177_13524585221097296 for Serum neurofilament light chain concentration predicts disease worsening in multiple sclerosis by Synne Brune, Einar A Høgestøl, Sigrid A de Rodez Benavent, Pål Berg-Hansen, Mona K Beyer, Ingvild Sørum Leikfoss, Steffan D Bos, Piotr Sowa, Cathrine Brunborg, Magi Andorra, Irene Pulido Valdeolivas, Susanna Asseyer, Alexander Brandt, Claudia Chien, Michael Scheel, Kaj Blennow, Henrik Zetterberg, Nicole Kerlero de Rosbo, Friedemann Paul, Antonio Uccelli, Pablo Villoslada, Tone Berge and Hanne F Harbo in Multiple Sclerosis Journal

sj-docx-12-msj-10.1177_13524585221097296 – Supplemental material for Serum neurofilament light chain concentration predicts disease worsening in multiple sclerosis Click here for additional data file. Supplemental material, sj-docx-12-msj-10.1177_13524585221097296 for Serum neurofilament light chain concentration predicts disease worsening in multiple sclerosis by Synne Brune, Einar A Høgestøl, Sigrid A de Rodez Benavent, Pål Berg-Hansen, Mona K Beyer, Ingvild Sørum Leikfoss, Steffan D Bos, Piotr Sowa, Cathrine Brunborg, Magi Andorra, Irene Pulido Valdeolivas, Susanna Asseyer, Alexander Brandt, Claudia Chien, Michael Scheel, Kaj Blennow, Henrik Zetterberg, Nicole Kerlero de Rosbo, Friedemann Paul, Antonio Uccelli, Pablo Villoslada, Tone Berge and Hanne F Harbo in Multiple Sclerosis Journal

sj-docx-13-msj-10.1177_13524585221097296 – Supplemental material for Serum neurofilament light chain concentration predicts disease worsening in multiple sclerosis Click here for additional data file. Supplemental material, sj-docx-13-msj-10.1177_13524585221097296 for Serum neurofilament light chain concentration predicts disease worsening in multiple sclerosis by Synne Brune, Einar A Høgestøl, Sigrid A de Rodez Benavent, Pål Berg-Hansen, Mona K Beyer, Ingvild Sørum Leikfoss, Steffan D Bos, Piotr Sowa, Cathrine Brunborg, Magi Andorra, Irene Pulido Valdeolivas, Susanna Asseyer, Alexander Brandt, Claudia Chien, Michael Scheel, Kaj Blennow, Henrik Zetterberg, Nicole Kerlero de Rosbo, Friedemann Paul, Antonio Uccelli, Pablo Villoslada, Tone Berge and Hanne F Harbo in Multiple Sclerosis Journal

sj-docx-14-msj-10.1177_13524585221097296 – Supplemental material for Serum neurofilament light chain concentration predicts disease worsening in multiple sclerosis Click here for additional data file. Supplemental material, sj-docx-14-msj-10.1177_13524585221097296 for Serum neurofilament light chain concentration predicts disease worsening in multiple sclerosis by Synne Brune, Einar A Høgestøl, Sigrid A de Rodez Benavent, Pål Berg-Hansen, Mona K Beyer, Ingvild Sørum Leikfoss, Steffan D Bos, Piotr Sowa, Cathrine Brunborg, Magi Andorra, Irene Pulido Valdeolivas, Susanna Asseyer, Alexander Brandt, Claudia Chien, Michael Scheel, Kaj Blennow, Henrik Zetterberg, Nicole Kerlero de Rosbo, Friedemann Paul, Antonio Uccelli, Pablo Villoslada, Tone Berge and Hanne F Harbo in Multiple Sclerosis Journal

sj-docx-15-msj-10.1177_13524585221097296 – Supplemental material for Serum neurofilament light chain concentration predicts disease worsening in multiple sclerosis Click here for additional data file. Supplemental material, sj-docx-15-msj-10.1177_13524585221097296 for Serum neurofilament light chain concentration predicts disease worsening in multiple sclerosis by Synne Brune, Einar A Høgestøl, Sigrid A de Rodez Benavent, Pål Berg-Hansen, Mona K Beyer, Ingvild Sørum Leikfoss, Steffan D Bos, Piotr Sowa, Cathrine Brunborg, Magi Andorra, Irene Pulido Valdeolivas, Susanna Asseyer, Alexander Brandt, Claudia Chien, Michael Scheel, Kaj Blennow, Henrik Zetterberg, Nicole Kerlero de Rosbo, Friedemann Paul, Antonio Uccelli, Pablo Villoslada, Tone Berge and Hanne F Harbo in Multiple Sclerosis Journal

sj-docx-16-msj-10.1177_13524585221097296 – Supplemental material for Serum neurofilament light chain concentration predicts disease worsening in multiple sclerosis Click here for additional data file. Supplemental material, sj-docx-16-msj-10.1177_13524585221097296 for Serum neurofilament light chain concentration predicts disease worsening in multiple sclerosis by Synne Brune, Einar A Høgestøl, Sigrid A de Rodez Benavent, Pål Berg-Hansen, Mona K Beyer, Ingvild Sørum Leikfoss, Steffan D Bos, Piotr Sowa, Cathrine Brunborg, Magi Andorra, Irene Pulido Valdeolivas, Susanna Asseyer, Alexander Brandt, Claudia Chien, Michael Scheel, Kaj Blennow, Henrik Zetterberg, Nicole Kerlero de Rosbo, Friedemann Paul, Antonio Uccelli, Pablo Villoslada, Tone Berge and Hanne F Harbo in Multiple Sclerosis Journal

sj-docx-17-msj-10.1177_13524585221097296 – Supplemental material for Serum neurofilament light chain concentration predicts disease worsening in multiple sclerosis Click here for additional data file. Supplemental material, sj-docx-17-msj-10.1177_13524585221097296 for Serum neurofilament light chain concentration predicts disease worsening in multiple sclerosis by Synne Brune, Einar A Høgestøl, Sigrid A de Rodez Benavent, Pål Berg-Hansen, Mona K Beyer, Ingvild Sørum Leikfoss, Steffan D Bos, Piotr Sowa, Cathrine Brunborg, Magi Andorra, Irene Pulido Valdeolivas, Susanna Asseyer, Alexander Brandt, Claudia Chien, Michael Scheel, Kaj Blennow, Henrik Zetterberg, Nicole Kerlero de Rosbo, Friedemann Paul, Antonio Uccelli, Pablo Villoslada, Tone Berge and Hanne F Harbo in Multiple Sclerosis Journal

sj-docx-18-msj-10.1177_13524585221097296 – Supplemental material for Serum neurofilament light chain concentration predicts disease worsening in multiple sclerosis Click here for additional data file. Supplemental material, sj-docx-18-msj-10.1177_13524585221097296 for Serum neurofilament light chain concentration predicts disease worsening in multiple sclerosis by Synne Brune, Einar A Høgestøl, Sigrid A de Rodez Benavent, Pål Berg-Hansen, Mona K Beyer, Ingvild Sørum Leikfoss, Steffan D Bos, Piotr Sowa, Cathrine Brunborg, Magi Andorra, Irene Pulido Valdeolivas, Susanna Asseyer, Alexander Brandt, Claudia Chien, Michael Scheel, Kaj Blennow, Henrik Zetterberg, Nicole Kerlero de Rosbo, Friedemann Paul, Antonio Uccelli, Pablo Villoslada, Tone Berge and Hanne F Harbo in Multiple Sclerosis Journal

sj-docx-19-msj-10.1177_13524585221097296 – Supplemental material for Serum neurofilament light chain concentration predicts disease worsening in multiple sclerosis Click here for additional data file. Supplemental material, sj-docx-19-msj-10.1177_13524585221097296 for Serum neurofilament light chain concentration predicts disease worsening in multiple sclerosis by Synne Brune, Einar A Høgestøl, Sigrid A de Rodez Benavent, Pål Berg-Hansen, Mona K Beyer, Ingvild Sørum Leikfoss, Steffan D Bos, Piotr Sowa, Cathrine Brunborg, Magi Andorra, Irene Pulido Valdeolivas, Susanna Asseyer, Alexander Brandt, Claudia Chien, Michael Scheel, Kaj Blennow, Henrik Zetterberg, Nicole Kerlero de Rosbo, Friedemann Paul, Antonio Uccelli, Pablo Villoslada, Tone Berge and Hanne F Harbo in Multiple Sclerosis Journal

sj-docx-8-msj-10.1177_13524585221097296 – Supplemental material for Serum neurofilament light chain concentration predicts disease worsening in multiple sclerosis Click here for additional data file. Supplemental material, sj-docx-8-msj-10.1177_13524585221097296 for Serum neurofilament light chain concentration predicts disease worsening in multiple sclerosis by Synne Brune, Einar A Høgestøl, Sigrid A de Rodez Benavent, Pål Berg-Hansen, Mona K Beyer, Ingvild Sørum Leikfoss, Steffan D Bos, Piotr Sowa, Cathrine Brunborg, Magi Andorra, Irene Pulido Valdeolivas, Susanna Asseyer, Alexander Brandt, Claudia Chien, Michael Scheel, Kaj Blennow, Henrik Zetterberg, Nicole Kerlero de Rosbo, Friedemann Paul, Antonio Uccelli, Pablo Villoslada, Tone Berge and Hanne F Harbo in Multiple Sclerosis Journal

sj-docx-9-msj-10.1177_13524585221097296 – Supplemental material for Serum neurofilament light chain concentration predicts disease worsening in multiple sclerosis Click here for additional data file. Supplemental material, sj-docx-9-msj-10.1177_13524585221097296 for Serum neurofilament light chain concentration predicts disease worsening in multiple sclerosis by Synne Brune, Einar A Høgestøl, Sigrid A de Rodez Benavent, Pål Berg-Hansen, Mona K Beyer, Ingvild Sørum Leikfoss, Steffan D Bos, Piotr Sowa, Cathrine Brunborg, Magi Andorra, Irene Pulido Valdeolivas, Susanna Asseyer, Alexander Brandt, Claudia Chien, Michael Scheel, Kaj Blennow, Henrik Zetterberg, Nicole Kerlero de Rosbo, Friedemann Paul, Antonio Uccelli, Pablo Villoslada, Tone Berge and Hanne F Harbo in Multiple Sclerosis Journal

sj-pdf-1-msj-10.1177_13524585221097296 – Supplemental material for Serum neurofilament light chain concentration predicts disease worsening in multiple sclerosis Click here for additional data file. Supplemental material, sj-pdf-1-msj-10.1177_13524585221097296 for Serum neurofilament light chain concentration predicts disease worsening in multiple sclerosis by Synne Brune, Einar A Høgestøl, Sigrid A de Rodez Benavent, Pål Berg-Hansen, Mona K Beyer, Ingvild Sørum Leikfoss, Steffan D Bos, Piotr Sowa, Cathrine Brunborg, Magi Andorra, Irene Pulido Valdeolivas, Susanna Asseyer, Alexander Brandt, Claudia Chien, Michael Scheel, Kaj Blennow, Henrik Zetterberg, Nicole Kerlero de Rosbo, Friedemann Paul, Antonio Uccelli, Pablo Villoslada, Tone Berge and Hanne F Harbo in Multiple Sclerosis Journal

sj-pdf-2-msj-10.1177_13524585221097296 – Supplemental material for Serum neurofilament light chain concentration predicts disease worsening in multiple sclerosis Click here for additional data file. Supplemental material, sj-pdf-2-msj-10.1177_13524585221097296 for Serum neurofilament light chain concentration predicts disease worsening in multiple sclerosis by Synne Brune, Einar A Høgestøl, Sigrid A de Rodez Benavent, Pål Berg-Hansen, Mona K Beyer, Ingvild Sørum Leikfoss, Steffan D Bos, Piotr Sowa, Cathrine Brunborg, Magi Andorra, Irene Pulido Valdeolivas, Susanna Asseyer, Alexander Brandt, Claudia Chien, Michael Scheel, Kaj Blennow, Henrik Zetterberg, Nicole Kerlero de Rosbo, Friedemann Paul, Antonio Uccelli, Pablo Villoslada, Tone Berge and Hanne F Harbo in Multiple Sclerosis Journal

sj-pdf-3-msj-10.1177_13524585221097296 – Supplemental material for Serum neurofilament light chain concentration predicts disease worsening in multiple sclerosis Click here for additional data file. Supplemental material, sj-pdf-3-msj-10.1177_13524585221097296 for Serum neurofilament light chain concentration predicts disease worsening in multiple sclerosis by Synne Brune, Einar A Høgestøl, Sigrid A de Rodez Benavent, Pål Berg-Hansen, Mona K Beyer, Ingvild Sørum Leikfoss, Steffan D Bos, Piotr Sowa, Cathrine Brunborg, Magi Andorra, Irene Pulido Valdeolivas, Susanna Asseyer, Alexander Brandt, Claudia Chien, Michael Scheel, Kaj Blennow, Henrik Zetterberg, Nicole Kerlero de Rosbo, Friedemann Paul, Antonio Uccelli, Pablo Villoslada, Tone Berge and Hanne F Harbo in Multiple Sclerosis Journal

sj-pdf-4-msj-10.1177_13524585221097296 – Supplemental material for Serum neurofilament light chain concentration predicts disease worsening in multiple sclerosis Click here for additional data file. Supplemental material, sj-pdf-4-msj-10.1177_13524585221097296 for Serum neurofilament light chain concentration predicts disease worsening in multiple sclerosis by Synne Brune, Einar A Høgestøl, Sigrid A de Rodez Benavent, Pål Berg-Hansen, Mona K Beyer, Ingvild Sørum Leikfoss, Steffan D Bos, Piotr Sowa, Cathrine Brunborg, Magi Andorra, Irene Pulido Valdeolivas, Susanna Asseyer, Alexander Brandt, Claudia Chien, Michael Scheel, Kaj Blennow, Henrik Zetterberg, Nicole Kerlero de Rosbo, Friedemann Paul, Antonio Uccelli, Pablo Villoslada, Tone Berge and Hanne F Harbo in Multiple Sclerosis Journal

sj-tiff-5-msj-10.1177_13524585221097296 – Supplemental material for Serum neurofilament light chain concentration predicts disease worsening in multiple sclerosis Click here for additional data file. Supplemental material, sj-tiff-5-msj-10.1177_13524585221097296 for Serum neurofilament light chain concentration predicts disease worsening in multiple sclerosis by Synne Brune, Einar A Høgestøl, Sigrid A de Rodez Benavent, Pål Berg-Hansen, Mona K Beyer, Ingvild Sørum Leikfoss, Steffan D Bos, Piotr Sowa, Cathrine Brunborg, Magi Andorra, Irene Pulido Valdeolivas, Susanna Asseyer, Alexander Brandt, Claudia Chien, Michael Scheel, Kaj Blennow, Henrik Zetterberg, Nicole Kerlero de Rosbo, Friedemann Paul, Antonio Uccelli, Pablo Villoslada, Tone Berge and Hanne F Harbo in Multiple Sclerosis Journal

sj-tiff-6-msj-10.1177_13524585221097296 – Supplemental material for Serum neurofilament light chain concentration predicts disease worsening in multiple sclerosis Click here for additional data file. Supplemental material, sj-tiff-6-msj-10.1177_13524585221097296 for Serum neurofilament light chain concentration predicts disease worsening in multiple sclerosis by Synne Brune, Einar A Høgestøl, Sigrid A de Rodez Benavent, Pål Berg-Hansen, Mona K Beyer, Ingvild Sørum Leikfoss, Steffan D Bos, Piotr Sowa, Cathrine Brunborg, Magi Andorra, Irene Pulido Valdeolivas, Susanna Asseyer, Alexander Brandt, Claudia Chien, Michael Scheel, Kaj Blennow, Henrik Zetterberg, Nicole Kerlero de Rosbo, Friedemann Paul, Antonio Uccelli, Pablo Villoslada, Tone Berge and Hanne F Harbo in Multiple Sclerosis Journal

sj-tiff-7-msj-10.1177_13524585221097296 – Supplemental material for Serum neurofilament light chain concentration predicts disease worsening in multiple sclerosis Click here for additional data file. Supplemental material, sj-tiff-7-msj-10.1177_13524585221097296 for Serum neurofilament light chain concentration predicts disease worsening in multiple sclerosis by Synne Brune, Einar A Høgestøl, Sigrid A de Rodez Benavent, Pål Berg-Hansen, Mona K Beyer, Ingvild Sørum Leikfoss, Steffan D Bos, Piotr Sowa, Cathrine Brunborg, Magi Andorra, Irene Pulido Valdeolivas, Susanna Asseyer, Alexander Brandt, Claudia Chien, Michael Scheel, Kaj Blennow, Henrik Zetterberg, Nicole Kerlero de Rosbo, Friedemann Paul, Antonio Uccelli, Pablo Villoslada, Tone Berge and Hanne F Harbo in Multiple Sclerosis Journal
