# Supplementary material for: Serum neurofilament light chain concentration predicts disease worsening in multiple sclerosis
Source: Mult Scler. 2022 Jun 4;28(12):1859–70. doi: 10.1177/13524585221097296 (PMC9493412; doi:10.1177/13524585221097296)
Supplement: sj-docx-10-msj-10.1177_13524585221097296 – Supplemental material for Serum neurofilament light chain concentration predicts disease worsening in multiple sclerosis [file sj-docx-10-msj-10.1177_13524585221097296.docx]

| **eTable 9** Univariable and multivariable models for disease worsening at two-year follow-up | | | | | | | | | |
| --- | --- | --- | --- | --- | --- | --- | --- | --- | --- |
|  | **Univariable analyses, age adjusted** | | | **Multivariable model 1 (n= 138)** | | | **Multivariable model 2 (n=188)** | | |
|  | OR | 95% CI | p-value | OR | 95% CI | p-value | OR | 95% CI | p-value |
| **sNfL ≥ 75 th*** | **1.8 (n=196)** | **1.00-3.12** | **0.050** | 1.94 | 0.92-4.1 | 0.083 | 1.75 | 0.95-3.22 | 0.073 |
| **Age (per 10 yrs)**** | 1.12 (n=196) | 0.84-1.49 | 0.455 | 0.903 | 0.56-1.46 | 0.677 | 0.79 | 0.53-1.19 | 0.257 |
| **Brain T2 Lesion volume (per SD)** | 1.09 (n=195) | 0.80-1.48 | 0.59 | 1.206 | 0.72-2.03 | 0.483 | 0.94 | 0.63-1.39 | 0.744 |
| **Normalized brain volume (per SD)** | 1.02 (n=194) | 0.73-1.44 | 0.89 | 1.023 | 0.62-1.7 | 0.93 | 0.93 | 0.60-1.43 | 0.732 |
| **GCIPL non-ON, continous** | 1.03 (n=154) | 0.99-1.07 | 0.161 | 1.05 | 1.00-1.11 | 0.051 |  |  |  |
| **pRNFL non-ON, continous** | 0.99 (n=147) | 0.97-1.02 | 0.67 | 0.97 | 0.94-1.04 | 0.13 |  |  |  |
| **9-HPT (per SD)** | **1.62 (n=190)** | **1.13-2.33** | **0.009** | **1.83** | **1.12-3.00** | **0.016** | **1.671** | **1.14-2.46** | **0.009** |
| **No treatment** | 1 |  |  | 1 |  |  | 1 |  |  |
| **Active treatment** | 0.55 (n=196) | 0.27-1.13 | 0.103 | **0.37** | **0.14-0.97** | **0.042** | 0.48 | 0.22-1.02 | 0.056 |
| **Highly active treatment** | 0.46 (n=196) | 0.21-1.0 | 0.056 | 0.345 | 0.12-1.03 | 0.057 | **0.38** | **0.16-0.92** | **0.032** |
| Abbreviations: sNfL= serum neurofilament light chain; GCIPL non-ON= ganglion cell innerplexiform layer in nonoptic neuritis eye; pRNFL non-ON = peripapillary retinal nervefiber layer thickness in nonoptic neuritis eye; 9-HPT= 9HolePegTest. *Cut off 75 th percentile: 20-29 years ≥ 4.325 pg/ml, 30-34 years ≥ 6.60 pg/ml, 35-39 years ≥ 7.05 pg/ml, 40-44 years ≥ 6.825 pg/ml, 45-49 years ≥ 7.075 pg/ml, 50-54 years ≥ 8.225 pg/ml, 55-59 years ≥ 11.15 pg/ml, 60-69 years ≥ 12.95 pg/ml. ** = not age adjusted. Results are presented with odds ratio (OR), 95% confidence interval (CI) and p-value. For treatment, no treatment is used as a reference category with OR=1. In bold are shown significant p-values, and the corresponding OR. P-values were not adjusted for multiple testing.   Univariable analyses, age adjusted Multivariabel model 1: whith OCT measures  Multivariabel model 2: whithout OCT measures | | | | | | | | | |
