# Supplementary material for: Serum neurofilament light chain concentration predicts disease worsening in multiple sclerosis
Source: Mult Scler. 2022 Jun 4;28(12):1859–70. doi: 10.1177/13524585221097296 (PMC9493412; doi:10.1177/13524585221097296)
Supplement: sj-docx-11-msj-10.1177_13524585221097296 – Supplemental material for Serum neurofilament light chain concentration predicts disease worsening in multiple sclerosis [file sj-docx-11-msj-10.1177_13524585221097296.docx]

| **eTable 8** Serum NfL percentile cut-offs as risk factors for disease worsening at two-year follow-up (n=196) | | | |
| --- | --- | --- | --- |
|  | **Disease worsening** | | |
|  | OR | 95% CI | p-value |
| **sNfL ≥ 75 th (NfL  ≥  8.0 pg/ml)** | **2.81** | **1.49-5.31** | **0.001** |
| **sNfL ≥ 80 th (NfL ≥  9.3 pg/ml)** | **1.98** | **1.02-3.83** | **0.043** |
| sNfL ≥ 85 th (NfL ≥  11.7 pg/ml) | 1.81 | 0.84-3.89 | 0.128 |
| sNfL ≥ 90 th (NfL ≥  12.4 pg/ml) | 2.2 | 0.99-4.9 | 0.053 |
| Abbreviations:  sNfL= serum neurofilament light chain. Results are presented with odds ratio (OR), 95% confidence interval (CI) and p-value. P-values < 0.05 (age-adjusted univariable analyses) are considered significant and are shown in bold together with the corresponding OR. P-values were not adjusted for multiple testing. | | | |
