# Supplementary material for: Serum neurofilament light chain concentration predicts disease worsening in multiple sclerosis
Source: Mult Scler. 2022 Jun 4;28(12):1859–70. doi: 10.1177/13524585221097296 (PMC9493412; doi:10.1177/13524585221097296)
Supplement: sj-docx-12-msj-10.1177_13524585221097296 – Supplemental material for Serum neurofilament light chain concentration predicts disease worsening in multiple sclerosis [file sj-docx-12-msj-10.1177_13524585221097296.docx]

| **eTable 7** Univariable and multivariable models for disease progression at two-year follow-up | | | | | | | | | |
| --- | --- | --- | --- | --- | --- | --- | --- | --- | --- |
|  | **Univariable analyses, age adjusted** | | | **Multivariable model 1 (n=138)** | | | **Multivariable model 2 (n=188)** | | |
|  | OR | 95% CI | p-value | OR | 95% CI | p-value | OR | 95% CI | p-value |
| **sNfL ≥ 75 th (NfL > 8 pg/ml)** | **2.8 (n=196)** | **1.49-5.31** | **0.001** | **3.23** | **1.37-7.57** | **0.007** | **2.8** | **1.4-5.6** | **0.004** |
| **Age*** | 1.01 (n=196) | 0.98-1.04 | 0.455 | 0.97 | 0.92-1.03 | 0.319 | 0.96 | 0.92-1.00 | 0.07 |
| **Brain T2 Lesion volume** | 1.01 (n=195) | 0.98-1.04 | 0.59 | 1.02 | 0.97-1.07 | 0.41 | 0.99 | 0.96-1.0 | 0.6 |
| **Normalized brain volume** | 1.00 (n=194) | 0.99-1.00 | 0.89 | 1.001 | 0.99-1.01 | 0.73 | 0.99 | 0.99-1.00 | 0.72 |
| **GCIPL non-ON ≥ 69 𝜇m** | 1.88 (n=154) | 0.94-3.72 | 0.072 | 2.15 | 0.92-5.02 | 0.078 |  |  |  |
| **pRNFL non-ON ≥ 103 𝜇m** | 0.86 (n=147) | 0.44-1.7 | 0.67 | 0.64 | 0.28-1.46 | 0.29 |  |  |  |
| **9-HPT** | **1.09 (n=190)** | **1.02-1.17** | **0.009** | **1.11** | **1.02-1.2** | **0.021** | **1.1** | **1.02-1.2** | **0.014** |
| **No treatment** | 1 |  |  | 1 |  |  | 1 |  |  |
| **Active treatment** | 0.55 (n=196) | 0.27-1.13 | 0.103 | 0.42 | 0.16-1.1 | 0.078 | 0.48 | 0.22-1.03 | 0.06 |
| **Highly active treatment** | 0.46 (n=196) | 0.21-1.0 | 0.056 | 0.35 | 0.11-1.05 | 0.061 | **0.37** | **0.15-0.91** | **0.03** |
| Abbreviations: sNfL= serum neurofilament light chain; GCIPL non-ON= ganglion cell innerplexiform layer in nonoptic neuritis eye; pRNFL non-ON = peripapillary retinal nervefiber layer thickness in nonoptic neuritis eye; 9-HPT= 9HolePegTest; * = not age adjusted .  Results are presented with odds ratio (OR), 95% confidence interval (CI) and p-value. For treatment, no treatment is used as a reference category with OR=1. In bold are shown significant p-values, and the corresponding OR. P-values were not adjusted for multiple testing.  Univariable analyses, age adjusted Multivariabel model 1: whith OCT measures  Multivariabel model 2: whithout OCT measures | | | | | | | | | |
