# Supplementary material for: Serum neurofilament light chain concentration predicts disease worsening in multiple sclerosis
Source: Mult Scler. 2022 Jun 4;28(12):1859–70. doi: 10.1177/13524585221097296 (PMC9493412; doi:10.1177/13524585221097296)
Supplement: sj-docx-13-msj-10.1177_13524585221097296 – Supplemental material for Serum neurofilament light chain concentration predicts disease worsening in multiple sclerosis [file sj-docx-13-msj-10.1177_13524585221097296.docx]

| **eTable 6**  Partial correlations between loge-sNfL concentrations and clinical, MRI and OCT measures | | | | |
| --- | --- | --- | --- | --- |
|  | **Baseline** | | **Two-year follow-up** | |
|  | **RRMS (n=257)** | **PMS (n=52)** | **RRMS (n=188)** | **PMS (n=38)** |
|  | **r_p_/p** | **r_p_/p** | **r_p_/p** | **r_p_/p** |
| **EDSS** | 0.07/ 0.25 | 0.24/ 0.11 | 0.14/ 0.07 | -0.12/ 0.50 |
| **9-HPT** | 0.12/ 0.06 | **0.38/ 0.01** | **0.24/ 0.003** | 0.28/ 0.14 |
| **25FWT** | -0.05/0.45 | 0.09/0.58 | **0.31/ <0.001** | 0.13/0.51 |
| **SDMT** | -0.01/ 0.92 | **-0.32/ 0.03** | -0.03/ 0.76 | -0.21/0.24 |
| **Brain T2 Lesion count** | **0.15/ 0.02** | **0.41/ 0.004** | 0.09/0.26 | **0.36/0.04** |
| **Brain T2 Lesion volume** | 0.06 / 0.32 | **0.39/ 0.01** | -0.06/0.47 | 0.26/0.15 |
| **Normalized brain volume** | 0.06/ 0.40 | -0.11/ 0.46 | -0.03/0.75 | -0.05/0.78 |
| **Normalized grey matter volume** | -0.10/0.12 | -0.07/0.65 | -0.09/0.29 | 0.04/0.85 |
| **Normalized white matter volume** | **0.13/0.04** | -0.08/0.58 | 0.04/0.61 | -0.12/0.52 |
| **Thalamus volume** | -0.01/ 0.83 | -0.14/0.34 | 0.07/0.39 | 0.09/0.70 |
| **pRNFL non-ON (𝜇m)** | -0.07/0.36 | 0.03/0.87 | 0.03/0.75 | 0.20/0.41 |
| **GCIPL non-ON (𝜇m)** | 0.08/0.24 | -0.02/0.94 | 0.07/0.45 | **-0.52/0.02** |
| **𝚫 EDSS** | 0.08/0.31 | -0.13/0.45 |  |  |
| **𝚫 9-HPT** | -0.05/0.565 | 0.17/0.34 |  |  |
| **𝚫 25FWT** | 0.07/0.38 | -0.004/0.98 |  |  |
| **𝚫 SDMT** | -0.09/0.28 | 0.07/0.66 |  |  |
| **𝚫 Brain T2 Lesion count** | **0.28/<0.001** | 0.02/0.91 |  |  |
| **𝚫 Brain T2 Lesion volume** | **0.21/0.01** | 0.07/0.68 |  |  |
| **𝚫 Normalized brain volume** | -0.11/0.18 | -0.10/0.56 |  |  |
| **𝚫 Normalized grey matter volume** | 0.01/0.89 | 0.09/0.58 |  |  |
| **𝚫 Normalized white matter volume** | -0.09/0.27 | -0.14/0.39 |  |  |
| **𝚫 Thalamus volume** | 0.01/0.88 | 0.10/0.59 |  |  |
| **𝚫 pRNFL non-ON (𝜇m)** | -0.15/0.07 | 0.08/0.73 |  |  |
| **𝚫 GCIPL non-ON (𝜇m)** | -0.15/0.07 | -0.22/0.33 |  |  |
| Abbreviations: HC= healthy control; RRMS = relapsing remitting MS; PMS = progressiv multiple scleriosis; sNfL= serum neurofilament light chain; OCT= optical coherence tomography; EDSS= expanded disability status scale; 9-HPT= 9HolePegTest; 25FWT= 25 foot walk test; SDMT= symbol digit modalities test; pRNFL non-ON = peripapillary retinal nervefiber layer thickness in nonoptic neuritis eye, GCIPL non-ON= ganglion cell innerplexiform layer in nonoptic neuritis eye; rp = partial correlation coefficient; 𝚫= change between baseline and two-year follow-up. Partial correlations coefficients are corrected for age, sex and treatment level. In bold are shown significant p-values, and the corresponding partial correlation coefficient. P-values were not adjusted for multiple testing. . | | | | |
