# Supplementary material for: Serum neurofilament light chain concentration predicts disease worsening in multiple sclerosis
Source: Mult Scler. 2022 Jun 4;28(12):1859–70. doi: 10.1177/13524585221097296 (PMC9493412; doi:10.1177/13524585221097296)
Supplement: sj-docx-14-msj-10.1177_13524585221097296 – Supplemental material for Serum neurofilament light chain concentration predicts disease worsening in multiple sclerosis [file sj-docx-14-msj-10.1177_13524585221097296.docx]

| **eTable 5** sNfL in subgroups of patients at baseline | | | | | | | |
| --- | --- | --- | --- | --- | --- | --- | --- |
|  | **HC (n=59)** | **RRMS no treatment (n=56)** | **RRMS active treatment (n=130)** | **RRMS higly active treatment (n=71)** | **PMS no treatment (n=34)** | **PMS active treatment (n= 7)** | **PMS higly active treatment (n=11)** |
| **sNfL (pg/ml) median (range)** | **6.0 (2.2-21.6) a** | 6.7 (3.2-20.6) | 6.7 (2.2-93.2) | 6.8 (3.9-33.3) | **11.3 (4.8-28.4) a** | 10.4 (8.6-14.0) | 9.5 (4.2-14.1) |
| **Age, years mean (SD)** | 39.9 (± 11.8) | 41.8 (± 7.8) | 41.8 (± 8.9) | 38.6 (± 8.3) | 55.7 (± 5.9) | 57.3 (± 5.9) | 47.2 (± 8.7) |
| **Sex, n (%)** |  |  |  |  |  |  |  |
| Female | 46 (78.0 %) | 43 (76.8 %) | 87 (66.9 %) | 56 (78.9 %) | 20 (58.8 %) | 4 (57.1 %) | 7 (63.6 %) |
| Abbreviations: HC= healthy control; RRMS = relapsing-remitting MS; PMS = progressive MS; sNfL= serum neurofilament light chain. Descriptive statistics are presented as either mean with standard deviation (SD), or median with range or proportions. p values were derived using linear regression models adjusted for age and sex. In bold are shown significant p values. No other significant correlations were seen between the subgroups of patients. P-values were not adjusted for multiple testing.  a PMS no treatment vs HC, p < 0.05, adjusted for age and sex. | | | | | | | |
