# Supplementary material for: Serum neurofilament light chain concentration predicts disease worsening in multiple sclerosis
Source: Mult Scler. 2022 Jun 4;28(12):1859–70. doi: 10.1177/13524585221097296 (PMC9493412; doi:10.1177/13524585221097296)
Supplement: sj-docx-15-msj-10.1177_13524585221097296 – Supplemental material for Serum neurofilament light chain concentration predicts disease worsening in multiple sclerosis [file sj-docx-15-msj-10.1177_13524585221097296.docx]

| **eTable 4** sNfL in healthy controls (HC) at baseline | | |
| --- | --- | --- |
|  | **HC (n=59)*** | **HC (n=309)**** |
| **sNfL (pg/ml) median (range)** | 6.0 (2.2-21.6) | 5.8 (1.1-33.0) |
| **Age, years mean (SD)** | 39.9 (± 11.8) | 42.6 (± 9.8) |
| **Sex, n (%)** |  |  |
| Female | 46 (78.0 %) | 217 (70.2 %) |
| Abbreviations: HC= healthy control; sNfL= serum neurofilament light chain. *Age and sex-matched healthy controls (HCs) collected at the four MS centres. **Age and sex-matched HC (Blennow and Zetterberg, unpublished). | | |
