# Supplementary material for: Serum neurofilament light chain concentration predicts disease worsening in multiple sclerosis
Source: Mult Scler. 2022 Jun 4;28(12):1859–70. doi: 10.1177/13524585221097296 (PMC9493412; doi:10.1177/13524585221097296)
Supplement: sj-docx-16-msj-10.1177_13524585221097296 – Supplemental material for Serum neurofilament light chain concentration predicts disease worsening in multiple sclerosis [file sj-docx-16-msj-10.1177_13524585221097296.docx]

| **eTable 3** Details regarding MRI acquisition | | | | |
| --- | --- | --- | --- | --- |
| **Center** | **Scanner** | **Vendor** | **Sequence** | **Details** |
| **Barcelona** | Tim Trio | Siemens Medical Systems, Erlangen, Germany | 3D MPRAGE | Resolution = 0.86 x 0.86 x 0.86 mm, TR = 1970 ms, TE = 2.41 ms |
|  |  |  | Axial T1-weighted post-gadolinium contrast agent sequence | Resolution = 0.31 x 0.31 x 3 mm, TR = 390 ms, TE = 2.65 ms |
|  |  |  | 3D FLAIR | Resolution = 1 x 1 x 1 mm, TR = 5000 ms, TE = 393 ms |
| **Oslo** | Discovery MR750 | GE Medical Systems | 3D BRAVO pre- and post-gadolinium | Resolution = 1 x 1 x 1 mm, TR = 8.16 ms, TE = 3.18 ms |
|  |  |  | 3D FLAIR | Resolution = 1 x 1 x 1.2 mm, TR = 8000 ms, TE = 127.25 ms |
| **Berlin** | Tim Trio | Siemens Medical Systems, Erlangen, Germany | 3D MPRAGE | Resolution = 1 x 1 x 1 mm, TR = 1900 ms, TE = 3.03 ms |
|  |  |  | 3D FLAIR | Resolution = 1 x 1 x 1 mm, TR = 6000 ms, TE = 388 ms |
| **Genova** | Signa HDxt | GE Medical Systems | FSPGD | Resolution = 1 x 1 x 1 mm, TR = 7.31 ms, TE = 3.00 ms |
|  |  |  | 3D TFE | Resolution = 1 x 1 x 1 mm, TR = 8.67 ms, TE = 4.00 ms |
|  |  |  | 3D FLAIR | Resolution = 1 x 1 x 1 mm, TR = 6000 ms, TE = 122.16 ms |
| Abbreviations: BRAVO; brain volume, FLAIR; fluid-attenuated inversion recovery, FSPGR; fast-spoiled gradient-echo, MPRAGE; magnetization prepared rapid gradient echo, TE; Echo Time, TFE; turbo field echo, TR; repetition time. Changes in scanners at follow-up: Barcelona and Genova; MAGNETOM Prisma, Siemens Medical Systems. | | | | |
