# Supplementary material for: Serum neurofilament light chain concentration predicts disease worsening in multiple sclerosis
Source: Mult Scler. 2022 Jun 4;28(12):1859–70. doi: 10.1177/13524585221097296 (PMC9493412; doi:10.1177/13524585221097296)
Supplement: sj-docx-17-msj-10.1177_13524585221097296 – Supplemental material for Serum neurofilament light chain concentration predicts disease worsening in multiple sclerosis [file sj-docx-17-msj-10.1177_13524585221097296.docx]

| **eTable 2** Age-normative cut-offs of sNfL calculated from HC data (n=309) | | | | | | | | |
| --- | --- | --- | --- | --- | --- | --- | --- | --- |
|  | **20-29 yrs (n=22)** | **30-34 yrs (n=55)** | **35-39 yrs (n=53)** | **40-44 yrs (n=50)** | **45-49 yrs (n=44)** | **50-54 yrs (n=46)** | **55-59 yrs (n=26)** | **60-69 yrs (n=13)** |
| **sNfL ≥ 75 th** | 4.33 | 6.60 | 7.05 | 6.83 | 7.08 | 8.23 | 11.15 | 12.95 |
| **sNfL ≥ 80 th** | 4.48 | 6.78 | 7.26 | 7.46 | 7.20 | 8.48 | 11.36 | 14.22 |
| **sNfL ≥ 85 th** | 4.66 | 7.62 | 7.68 | 7.70 | 7.33 | 9.18 | 12.16 | 15.34 |
| **sNfL ≥ 90 th** | 5.82 | 7.78 | 8.56 | 8.42 | 7.50 | 9.98 | 12.72 | 15.68 |
| Abbreviations: sNfL= serum neurofilament light chain, HC= healthy control. | | | | | | | | |
