# Supplementary material for: Serum neurofilament light chain concentration predicts disease worsening in multiple sclerosis
Source: Mult Scler. 2022 Jun 4;28(12):1859–70. doi: 10.1177/13524585221097296 (PMC9493412; doi:10.1177/13524585221097296)
Supplement: sj-docx-18-msj-10.1177_13524585221097296 – Supplemental material for Serum neurofilament light chain concentration predicts disease worsening in multiple sclerosis [file sj-docx-18-msj-10.1177_13524585221097296.docx]

| **eTable 1** Distribution of MS samples in each percentile category | |
| --- | --- |
|  | MS (n= 309) (%) |
| sNfL ≥ 25 th (NfL ≥ 4.4 pg/ml) | 280 (90.6%) |
| sNfL ≥ 50 th (NfL ≥ 6.0 pg/ml) | 206 (66.7%) |
| sNfL ≥ 75 th (NfL ≥ 8.0 pg/ml) | 133 (43%) |
| sNfL ≥ 80 th (NfL ≥ 9.3 pg/ml) | 101 (32.7%) |
| sNfL ≥ 85 th (NfL ≥ 11.7 pg/ml) | 54 (17.5%) |
| sNfL ≥ 90 th (NfL ≥ 12.4 pg/ml) | 48 (15.5 %) |
| sNfL ≥ 95 th (NfL ≥ 15.1 pg/ml) | 28 (9.1) |
| sNfL ≥ 97.5 th (NfL ≥ 19.2 pg/ml) | 11 (3.6%) |
| Abbreviations: sNfL= serum neurofilament light chain | |
