# Supplementary material for: Serum neurofilament light chain concentration predicts disease worsening in multiple sclerosis
Source: Mult Scler. 2022 Jun 4;28(12):1859–70. doi: 10.1177/13524585221097296 (PMC9493412; doi:10.1177/13524585221097296)
Supplement: sj-docx-19-msj-10.1177_13524585221097296 – Supplemental material for Serum neurofilament light chain concentration predicts disease worsening in multiple sclerosis [file sj-docx-19-msj-10.1177_13524585221097296.docx]

Supplemental data

## Materials and Methods

### Inclusion criteria

Inclusion criteria included clinically isolated syndrome or MS diagnosis based on the 2010 revision of McDonald criteria,^1^ and age 18-80 years. Exclusion criteria were presence of relapse or steroid treatment within 30 days before the study invitation, chronic diseases other than MS or pregnancy during the course of the study. Patients using DMTs needed to be stable for at least one year when treated with interferon beta or glatiramer acetate or at least six months for other treatments. For patients previously treated with DMTs, a washout of at least three months was required (six months for ocrelizumab or rituximab; one year following alemtuzumab).

### Serum neurofilament light chain measurements

Serum samples were collected with 4 mL Vacuette Z Serum Clot Activator Tube® (Greiner bio-one International) and processed within one hour by centrifugation at 2000*g* for 10 minutes at 4^o^C. Serum was aliquoted and immediately stored at -80^o^C until analysis. Measurement of NfL concentrations in serum samples from MS patients and HCs were performed in the Clinical Neurochemistry Laboratory at the Sahlgrenska University Hospital, Sweden, using the highly sensitive single molecule array immunoassay (Simoa Technology; QUANTERIX Corporation).^2^ A single batch of reagents was used and intra-assay coefficients of variation were below 10%.

### Optical coherence tomography

Retinal OCT scans were acquired both at baseline and at two-year follow-up by trained technicians, blinded to the patient’s clinical history. The scans were performed in eye-tracking mode, under standard ambient light conditions (lighting level of 80–100 foot-candles), without the use of pharmacologic dilatation. A Spectralis device or Nidek RS-3000 was used. The pRNFL thickness (μm) was measured with a 12 degree diameter ring scan automatically centered on the optic nerve head (100 ART, 1,536 A-Scans per B scan). The macular scan protocol involved a 20 x 20 degree horizontal raster scan centered on the fovea, including 25 B scans (ART ≥9, 512 A-Scans per B scan). A single grader at the reading center in Berlin performed intra-retinal layer segmentation, on the raw OCT data, using Orion software® (Voxeleron inc, Berkeley, US) to quantify the macular ganglion cell plus inner plexiform layer (GCIPL) and the macular inner nuclear layer (INL) thicknesses (μm) in the 6 mm ring area. All OCT scans fulfilled the OSCAR-IB criteria.^3^ Only OCT measures from eyes with no previous history of optic neuritis at baseline were included in the analyses. When patients had both eyes eligible for inclusion, the number of observations were reduced to one per patient, randomly including analyses of the left eye in half of the patients and the right eye in the other half. The patients were grouped by a median split into those with a thin (<69 𝜇m) or a thick GCIPL (≥ 69 𝜇m), and a thin (<103 𝜇m) or a thick peripapillary retinal-nerve fiber layer (pRNFL) (≥103 𝜇m).

### **MRI acquisition and structural MRI pre-and post-processing**

All MRI acquisitions were harmonized across all four centers with minimum requirements for MRI scanners and sequences. All analyses of MRI data were performed at the Berlin reading centre according to a unified pipeline including alignment to Montreal Neurological Institute (MNI)-152 standard space (using fslreorient2std), white and grey matter brain masking (Computational Anatomy Toolbox 12 Toolbox for MATLAB SPM12), N4-bias field correction (Advanced Normalization Tools, http://stnava.github.io/ANTs/) and linear, rigid body registration of T2-weighted (FLAIR) images to T1-weighted (MPRAGE, BRAVO, and FSPGR) images (FSL FLIRT). T1-weighted, FLAIR and post-contrast agent T1-weighted follow-up scans were co-registered to each individual first session of each patient. T2-hyperintense lesion segmentation was performed using ITK-SNAP ([www.itksnap.org](http://www.itksnap.org)) by two experienced MRI technicians, where changes in lesions were marked by overlaying the follow-up on the first session scans. Lesion counts and volumes were extracted from lesion masks using fslmaths (<https://fsl.fmrib.ox.ac.uk/fsl/fslwiki/Fslutils>). Lesion filling on T1-weighted images was performed using the FSL lesion filling tool (<https://fsl.fmrib.ox.ac.uk/fsl/fslwiki/lesion_filling>), utilizing white matter masks created from the Computational Anatomy Toolbox for SPM12 (CAT12, <http://www.neuro.uni-jena.de/cat/>). The resulting lesion-filled T1-weighted images were then used for whole-brain white and grey matter volume extraction, including the follow-up session percentage brain tissue volume change (PBVC), normalised for subject head size, was estimated with SIENAX, part of FSL.

### Statistical analysis

OCT measures were missing in 27% of the patients, and to investigate the possible uncertainty due to missing data in the multivariable logistic regression model with OCT measures, sensitivity analysis of imputation were performed. In the multivariable analyses including dichotomous OCT variables, a best and worst case scenario sensitivity analysis was performed. In the sensitivity analyses, missing values for GCIPL and pRNFL were thus first imputed by having thinner (worst) and secondly imputed by not having thinner (best) measures. Then the model was re-analysed for those imputed with worst and best imputed OCT measures in separate regression analyses.^4^ In the multivariable analyses including OCT data as continous variables, missing values in covariates were imputed using multiple imputation as a sensitivity analysis. For multiple imputation all available data was used to generate 30 imputed data sets. The results from each complete data set were combined to present single estimates.

## Results:

To analyze whether sNfL, MRI and OCT parameters combined showed stronger associations with disease worsening than each parameter alone, we combined sNfL, MRI and OCT measures in multivariable models. In the first multivariable analysis (multivariable model 1) including the OCT data (supplementary eTable 7), the significant association between sNfL concentration and disease worsening was even more pronounced (OR [95 % CI] = 3.2, [1.4-7.6], p= 0.007). After imputing missing OCT measures, as a sensitivity analysis, the sNfL concentrations still correlated significantly with disease worsening (OR [95 % CI] = 2.7, [1.3-5,4], p= 0.006 best case, OR [95 % CI] = 2.8, [1.3-5,6], p= 0.004 worst case). Since OCT data were available for a subgroup of the patients (73%), a second model (multivariable model 2) using multivariable analyses without OCT data was applied. The results from this second model showed that high sNfL concentrations at baseline were significantly associated with disease worsening at follow-up (OR [95 % CI] = 2.8, [1.4-5.6], p=0.004). Neither GCIPL, pRNFL, T2 lesion volume nor normalized brain volume measures at baseline were associated with disease worsening at follow-up in either of the multivariable models. Slower performance on 9-HPT was also significantly associated with disease worsening at follow-up in both multivariable model 1 and 2. The data indicated that patients with active or highly active treatment had less disease worsening compared to untreated patients.

In the multivariable analysis, combining age-normative cut-offs of sNfL and OCT measures at continuous variables (supplementary eTable 9), there was a trend towards a positive association between high sNfL concentration and disease worsening, although this result was not significant. After imputing missing OCT measures, using multiple imputation as a sensitivity analysis, the sNfL concentrations showed no association with disease worsening (OR [95 % CI] = 1.55, [0.84-2.84], p= 0.158).

1. Polman CH, Reingold SC, Banwell B, et al. Diagnostic criteria for multiple sclerosis: 2010 revisions to the McDonald criteria. *Ann Neurol* 2011; 69: 292-302. 2011/03/10. DOI: 10.1002/ana.22366.

2. Disanto G, Barro C, Benkert P, et al. Serum Neurofilament light: A biomarker of neuronal damage in multiple sclerosis. *Ann Neurol* 2017; 81: 857-870. 2017/05/18. DOI: 10.1002/ana.24954.

3. Schippling S, Balk LJ, Costello F, et al. Quality control for retinal OCT in multiple sclerosis: validation of the OSCAR-IB criteria. *Mult Scler* 2015; 21: 163-170. 2014/06/21. DOI: 10.1177/1352458514538110.

4. Pedersen AB, Mikkelsen EM, Cronin-Fenton D, et al. Missing data and multiple imputation in clinical epidemiological research. *Clin Epidemiol* 2017; 9: 157-166. 2017/03/30. DOI: 10.2147/clep.S129785.
