# Supplementary material for: Serum neurofilament light chain concentration predicts disease worsening in multiple sclerosis
Source: Mult Scler. 2022 Jun 4;28(12):1859–70. doi: 10.1177/13524585221097296 (PMC9493412; doi:10.1177/13524585221097296)
Supplement: sj-docx-8-msj-10.1177_13524585221097296 – Supplemental material for Serum neurofilament light chain concentration predicts disease worsening in multiple sclerosis [file sj-docx-8-msj-10.1177_13524585221097296.docx]

**Supplementary figure legends**

Supplementary eFigure 1: A histogram illustrating the variability of the follow-up duration in the patient population.

Supplementary eFigure 2: Scatterplots depicting sNfL values by age separately presented for HCs (a) and MS patients (b). sNfL= serum neurofilament light chain; RRMS= relapsing remitting multiple sclerosis; PMS = progressive multiple sclerosis.

Supplementary eFigure 3: A boxplot depicting sNfL concentrations in subgroups of patients at baseline. HC= healthy control; RRMS = relapsing-remitting MS; PMS = progressive MS; sNfL= serum neurofilament light chain. PMS no treatment vs HC, p < 0.05, adjusted for age and sex. P-values were not adjusted for multiple testing.
