# Supplementary material for: Serum neurofilament light chain concentration predicts disease worsening in multiple sclerosis
Source: Mult Scler. 2022 Jun 4;28(12):1859–70. doi: 10.1177/13524585221097296 (PMC9493412; doi:10.1177/13524585221097296)
Supplement: sj-docx-9-msj-10.1177_13524585221097296 – Supplemental material for Serum neurofilament light chain concentration predicts disease worsening in multiple sclerosis [file sj-docx-9-msj-10.1177_13524585221097296.docx]

| **eTable 10** Serum NfL percentile cut-offs as risk factors for disease worsening, or its components, at two-year follow-up | | | | | | | | | | | | |
| --- | --- | --- | --- | --- | --- | --- | --- | --- | --- | --- | --- | --- |
|  | **Disease worsening (n=196)** | | | **≥ 3 new cerebral MRI lesions (n=205)** | | | **EDSS progression (n=206)** | | | **Relapse (n=177)** | | |
|  | OR | 95% CI | p-value | OR | 95% CI | p-value | OR | 95% CI | p-value | OR | 95% CI | p-value |
| **sNfL ≥ 75 th *** | **1.77** | **1.00-3.12** | **0.050** | **2.29** | **1.07-4.93** | **0.034** | 1.201 | 0.598-2.413 | 0.607 | 1.978 | 0.914-4.281 | 0.083 |
| **sNfL ≥ 80 th **** | **2.14** | **1.20-3.79** | **0.009** | **2.58** | **1.20-5.56** | **0.015** | 1.355 | 0.674-2.725 | 0.393 | **2.281** | **1.502-4.945** | **0.037** |
| **sNfL ≥ 85 th ***** | **2.28** | **1.27-4.06** | **0.005** | **3.22** | **1.49-6.96** | **0.003** | 1.491 | 0.74-3.004 | 0.264 | **3.223** | **1.493-6.957** | **0.003** |
| **sNfL ≥ 90 th ****** | **2.35** | **1.29-4.29** | **0.005** | **3.66** | **1.71-7.83** | **0.001** | 1.538 | 0.751-3.152 | 0.239 | 2.093 | 0.965-4.544 | 0.062 |
| Abbreviations: sNfL= serum neurofilament light chain. Results are presented with odds ratio (OR), 95% confidence interval (CI) and p-value. In bold are shown significant p-values, and the corresponding OR. P-values were not adjusted for multiple testing *Cut off 75 th percentile: 20-29 years ≥ 4.325 pg/ml, 30-34 years ≥ 6.60 pg/ml, 35-39 years ≥ 7.05 pg/ml, 40-44 years ≥ 6.825 pg/ml, 45-49 years ≥ 7.075 pg/ml, 50-54 years ≥ 8.225 pg/ml, 55-59 years ≥ 11.15 pg/ml, 60-69 years ≥ 12.95 pg/ml. **Cut off 80 th percentile: 20-29 years ≥ 4.48 pg/ml, 30-34 years ≥ 6.78 pg/ml, 35-39 years ≥ 7.26 pg/ml, 40-44 years ≥ 7.46 pg/ml, 45-49 years ≥ 7.2 pg/ml, 50-54 years ≥ 8.48 pg/ml, 55-59 years ≥ 11.36 pg/ml, 60-69 years ≥ 14.22 pg/ml. ***Cut off 85 th percentile: 20-29 years ≥ 4.65 pg/ml, 30-34 years ≥ 7.62 pg/ml, 35-39 years ≥ 7.68 pg/ml, 40-44 years ≥ 7.7 pg/ml, 45-49 years ≥ 7.325 pg/ml, 50-54 years ≥ 9.175 pg/ml, 55-59 years ≥ 12.160 pg/ml, 60-69 years ≥ 15.34 pg/ml. ****Cut off 90 th percentile: 20-29 years ≥ 5.82pg/ml, 30-34 years ≥ 7.78 pg/ml, 35-39 years ≥ 8.56 pg/ml, 40-44 years ≥ 8.42 pg/ml, 45-49 years ≥ 7.5 pg/ml, 50-54 years ≥ 9.98 pg/ml, 55-59 years ≥ 12.72 pg/ml, 60-69 years ≥ 15.68 pg/ml. | | | | | | | | | | | | |
