# Supplementary material for: Serum neurofilament light chain concentration predicts disease worsening in multiple sclerosis
Source: Mult Scler. 2022 Jun 4;28(12):1859–70. doi: 10.1177/13524585221097296 (PMC9493412; doi:10.1177/13524585221097296)
Supplement: sj-pdf-1-msj-10.1177_13524585221097296 – Supplemental material for Serum neurofilament light chain concentration predicts disease worsening in multiple sclerosis [file sj-pdf-1-msj-10.1177_13524585221097296.pdf]

# MS Journal Appendix for MRI methodology

| Hardware | |
|---|---|
| Field strength | 3 Tesla |
| Manufacturer | Siemens |
| Model | TimTrio |
| Coil type<br>(e.g. head, surface) | Head |
| Number of coil channels | |

| Acquisition sequence | | |
|---|---|---|
| Type<br>(e.g. FLAIR, DIR, DTI, fMRI) | MPRAGE, FLAIR | |
| Acquisition time | MPRAGE: 5:36 min; FLAIR: 6:30 min | |
| Orientation | HFS | |
| Alignment<br>(e.g. anterior commissure/poster commissure line) | | |
| Voxel size | MPRAGE & FLAIR: 0.9 mm isotropic | |
| TR | MPRAGE: 1970 ms; FLAIR: 5000 ms | |
| TE | MPRAGE: 2.51 ms; FLAIR: 303 ms | |
| TI | MPRAGE: 1050 ms; FLAIR: 1800 ms | |
| Flip angle | MPRAGE: 9°; FLAIR: 120° | |
| NEX | MPRAGE: 1; FLAIR: 1 | |
| Field of view | MPRAGE & FLAIR: 256x256x208 | |
| Matrix size | MPRAGE & FLAIR: 256x256 | |
| Parallel imaging | Yes | No (circled) |
| If used, parallel imaging method:<br>(e.g. SENSE, GRAPPA) | | |
| Cardiac gating | Yes | No (circled) |
| If used, cardiac gating method:<br>(e.g. PPU or ECG) | | |
| Contrast enhancement | Yes (circled) | No (circled) |

| Acquisition sequence | |
|---|---|
| If used, provide name of contrast agent, dose and timing of scan post-contrast administration | |
| Other parameters: | |

| Image analysis methods and outputs | |
|---|---|
| ***Lesions*** | |
| Type<br>(e.g. Gd-enhancing, T2-hyperintense, T1-hypointense) | |
| Analysis method | |
| Analysis software | |
| Output measure<br>(e.g. count or volume [ml]) | |
| ***Tissue volumes*** | |
| Type<br>(e.g. whole brain, grey matter, white matter, spinal cord) | |
| Analysis method | |
| Analysis software | |
| Output measure<br>(e.g. absolute tissue volume in ml, tissue volume as a fraction of intracranial volume, percentage change in tissue volumes) | |
| ***Tissue measures (e.g. MTR, DTI, T1-RT, T2-RT, T2*, T2', $^{1}$H-MRS, perfusion, Na)*** | |
| Type<br>(e.g. whole brain, grey matter, white matter, spinal cord, normal-appearing grey matter or white matter) | |
| Analysis method | |
| Analysis software | |
| Output measure | |
| ***Other MRI measures (e.g. functional MRI)*** | |
| Type<br>(e.g. whole brain, grey matter, white matter, spinal cord, normal-appearing grey matter or white matter) | |
| Analysis method | |
| Analysis software | |
| Output measure | |

**Other analysis details:**
