# Supplementary material for: Serum neurofilament light chain concentration predicts disease worsening in multiple sclerosis
Source: Mult Scler. 2022 Jun 4;28(12):1859–70. doi: 10.1177/13524585221097296 (PMC9493412; doi:10.1177/13524585221097296)
Supplement: sj-pdf-2-msj-10.1177_13524585221097296 – Supplemental material for Serum neurofilament light chain concentration predicts disease worsening in multiple sclerosis [file sj-pdf-2-msj-10.1177_13524585221097296.pdf]

MS Journal Appendix for MRI methodology

Hardware	
Field strength	3 Tesla
Manufacturer	Siemens
Model	TimTrio
Coil type<br>(e.g. head, surface)	Head
Number of coil channels	32

Acquisition sequence		
Type<br>(e.g. FLAIR, DIR, DTI, fMRI)	MPRAGE, FLAIR	
Acquisition time	MPRAGE: 4:24 min; FLAIR: 7:38 min	
Orientation	HFS	
Alignment<br>(e.g. anterior commissure/poster commissure line)	anterior to posterior commissure line	
Voxel size	MPRAGE & FLAIR: 1mm isotropic	
TR	MPRAGE: 1900 ms; FLAIR: 6000 ms	
TE	MPRAGE: 3.03 ms; FLAIR: 388 ms	
TI	MPRAGE: 900 ms; FLAIR: 2100 ms	
Flip angle	MPRAGE: 9°; FLAIR: 120°	
NEX	MPRAGE & FLAIR: 1	
Field of view	MPRAGE & FLAIR: 256x256x176	
Matrix size	MPRAGE & FLAIR: 256x256	
Parallel imaging	Yes	No (circled)
If used, parallel imaging method:<br>(e.g. SENSE, GRAPPA)		
Cardiac gating	Yes	No (circled)
If used, cardiac gating method:<br>(e.g. PPU or ECG)		
Contrast enhancement	Yes	No (circled)

Acquisition sequence	
If used, provide name of contrast agent, dose and timing of scan post-contrast administration	
Other parameters:	

Image analysis methods and outputs	
Lesions	
Type<br>(e.g. Gd-enhancing, T2-hyperintense, T1-hypointense)	
Analysis method	
Analysis software	
Output measure<br>(e.g. count or volume [ml])	
Tissue volumes	
Type<br>(e.g. whole brain, grey matter, white matter, spinal cord)	
Analysis method	
Analysis software	
Output measure<br>(e.g. absolute tissue volume in ml, tissue volume as a fraction of intracranial volume, percentage change in tissue volumes)	
Tissue measures (e.g. MTR, DTI, T1-RT, T2-RT, T2*, T2', ^{1}H-MRS, perfusion, Na)	
Type<br>(e.g. whole brain, grey matter, white matter, spinal cord, normal-appearing grey matter or white matter)	
Analysis method	
Analysis software	
Output measure	
Other MRI measures (e.g. functional MRI)	
Type<br>(e.g. whole brain, grey matter, white matter, spinal cord, normal-appearing grey matter or white matter)	
Analysis method	
Analysis software	
Output measure	

Other analysis details:
