# Supplementary material for: Serum neurofilament light chain concentration predicts disease worsening in multiple sclerosis
Source: Mult Scler. 2022 Jun 4;28(12):1859–70. doi: 10.1177/13524585221097296 (PMC9493412; doi:10.1177/13524585221097296)
Supplement: sj-pdf-3-msj-10.1177_13524585221097296 – Supplemental material for Serum neurofilament light chain concentration predicts disease worsening in multiple sclerosis [file sj-pdf-3-msj-10.1177_13524585221097296.pdf]

# MS Journal Appendix for MRI methodology

| Hardware | |
|---|---|
| Field strength | 3 Tesla |
| Manufacturer | Siemens & GE |
| Model | Prisma & Sygna HDxt |
| Coil type (e.g. head, surface) | Head |
| Number of coil channels | |

| Acquisition sequence | | |
|---|---|---|
| Type (e.g. FLAIR, DIR, DTI, fMRI) | GE: FSPGR, FLAIR CUBE; Siemens: MPRAGE, FLAIR | |
| Acquisition time | MPRAGE: 5:09 min; FLAIR: 7:38 min | |
| Orientation | HFS | |
| Alignment (e.g. anterior commissure/poster commissure line) | | |
| Voxel size | FLAIR CUBE: 0.5x0.5x1mm; FSPGR, MPRAGE, FLAIR: 1mm isotropic | |
| TR | FLAIR CUBE: 6000 ms; FSPGR:7.312 ms; MPRAGE: 2300 ms; FLAIR: 6000 ms | |
| TE | FLAIR CUBE: 122.162 ms; FSPGR: 2.996 ms; MPRAGE: 2.96 ms; FLAIR: 388 ms | |
| TI | FLAIR CUBE: 1872 ms; FSPGR: 0 ms; MPRAGE: 919 ms; FLAIR: 2100 ms | |
| Flip angle | FLAIR CUBE: 90°; FSPGR: 15°; MPRAGE: 8°; FLAIR: 120° | |
| NEX | FLAIR CUBE, FSPGR, MPRAGE, FLAIR: 1 | |
| Field of view | FLAIR CUBE: 512x512x176; FSPGR: 256x256x172; MPRAGE & FLAIR: 256x256x176 | |
| Matrix size | FLAIR CUBE: 512x512; FSPGR, MPRAGE, FLAIR: 256x256 | |
| Parallel imaging | Yes | No (circled) |
| If used, parallel imaging method: (e.g. SENSE, GRAPPA) | | |
| Cardiac gating | Yes | No (circled) |
| If used, cardiac gating method: (e.g. PPU or ECG) | | |
| Contrast enhancement | Yes (circled) | No (circled) |

| Acquisition sequence | |
|---|---|
| If used, provide name of contrast agent, dose and timing of scan post-contrast administration | |
| Other parameters: | |

| Image analysis methods and outputs | |
|---|---|
| ***Lesions*** | |
| Type<br>(e.g. Gd-enhancing, T2-hyperintense, T1-hypointense) | |
| Analysis method | |
| Analysis software | |
| Output measure<br>(e.g. count or volume [ml]) | |
| ***Tissue volumes*** | |
| Type<br>(e.g. whole brain, grey matter, white matter, spinal cord) | |
| Analysis method | |
| Analysis software | |
| Output measure<br>(e.g. absolute tissue volume in ml, tissue volume as a fraction of intracranial volume, percentage change in tissue volumes) | |
| ***Tissue measures (e.g. MTR, DTI, T1-RT, T2-RT, T2*, T2', $^{1}$H-MRS, perfusion, Na)*** | |
| Type<br>(e.g. whole brain, grey matter, white matter, spinal cord, normal-appearing grey matter or white matter) | |
| Analysis method | |
| Analysis software | |
| Output measure | |
| ***Other MRI measures (e.g. functional MRI)*** | |
| Type<br>(e.g. whole brain, grey matter, white matter, spinal cord, normal-appearing grey matter or white matter) | |
| Analysis method | |
| Analysis software | |
| Output measure | |

**Other analysis details:**
