# Supplementary material for: Serum neurofilament light chain concentration predicts disease worsening in multiple sclerosis
Source: Mult Scler. 2022 Jun 4;28(12):1859–70. doi: 10.1177/13524585221097296 (PMC9493412; doi:10.1177/13524585221097296)
Supplement: sj-pdf-4-msj-10.1177_13524585221097296 – Supplemental material for Serum neurofilament light chain concentration predicts disease worsening in multiple sclerosis [file sj-pdf-4-msj-10.1177_13524585221097296.pdf]

MS Journal Appendix for MRI methodology

Hardware	
Field strength	3T
Manufacturer	GE Medical Systems
Model	Discovery MR 750
Coil type<br>(e.g. head, surface)	Head
Number of coil channels	

Acquisition sequence		
Type<br>(e.g. FLAIR, DIR, DTI, fMRI)	3D BRAVO pre-and post-gadolinium, 3D FLAIR	
Acquisition time	In total about 75 minutes including spine	
Orientation		
Alignment<br>(e.g. anterior commissure/poster commissure line)		
Voxel size	3D BRAVO: 1.0x1.0x1.0. 3D FLAIR: 1.2x1.2x1.2	
TR	3D BRAVO: 8,16 ms. 3D FLAIR: 8000 ms	
TE	3D BRAVO: 3,18 ms. 3D FLAIR: 127,25 ms	
TI	3D BRAVO: 450 3D FLAIR 2240	
Flip angle	3D BRAVO: 12	
NEX	3D BRAVO 1.00. 3D FLAIR: 1.00	
Field of view	3D BRAVO: 25.6. 3D FLAIR 25.6	
Matrix size		
Parallel imaging	Yes	No
If used, parallel imaging method:<br>(e.g. SENSE, GRAPPA)		
Cardiac gating	Yes	No
If used, cardiac gating method:<br>(e.g. PPU or ECG)		
Contrast enhancement	**YES**	No

Acquisition sequence	
If used, provide name of contrast agent, dose and timing of scan post-contrast administration	Gadolinium , 0.2 ml/kg, Timing: 5 min
Other parameters:	DTI, RSI, rs-fMRI, medualla

Image analysis methods and outputs	
Lesions	
Type<br>(e.g. Gd-enhancing, T2-hyperintense, T1-hypointense)	T2-hyperintense & Gd-enhancing
Analysis method	See methods section and supplementary
Analysis software	See methods section and supplementary
Output measure<br>(e.g. count or volume [ml])	See methods section and supplementary
Tissue volumes	
Type<br>(e.g. whole brain, grey matter, white matter, spinal cord)	See methods section and supplementary
Analysis method	See methods section and supplementary
Analysis software	See methods section and supplementary
Output measure<br>(e.g. absolute tissue volume in ml, tissue volume as a fraction of intracranial volume, percentage change in tissue volumes)	See methods section and supplementary
Tissue measures (e.g. MTR, DTI, T1-RT, T2-RT, T2*, T2', ^{1}H-MRS, perfusion, Na)	
Type<br>(e.g. whole brain, grey matter, white matter, spinal cord, normal-appearing grey matter or white matter)	
Analysis method	
Analysis software	
Output measure	
Other MRI measures (e.g. functional MRI)	
Type<br>(e.g. whole brain, grey matter, white matter, spinal cord, normal-appearing grey matter or white matter)	
Analysis method	
Analysis software	
Output measure	

Other analysis details:
