# Supplementary figures and images for: Serum neurofilament light chain concentration predicts disease worsening in multiple sclerosis
Source: Mult Scler. 2022 Jun 4;28(12):1859–70. doi: 10.1177/13524585221097296 (PMC9493412; doi:10.1177/13524585221097296)

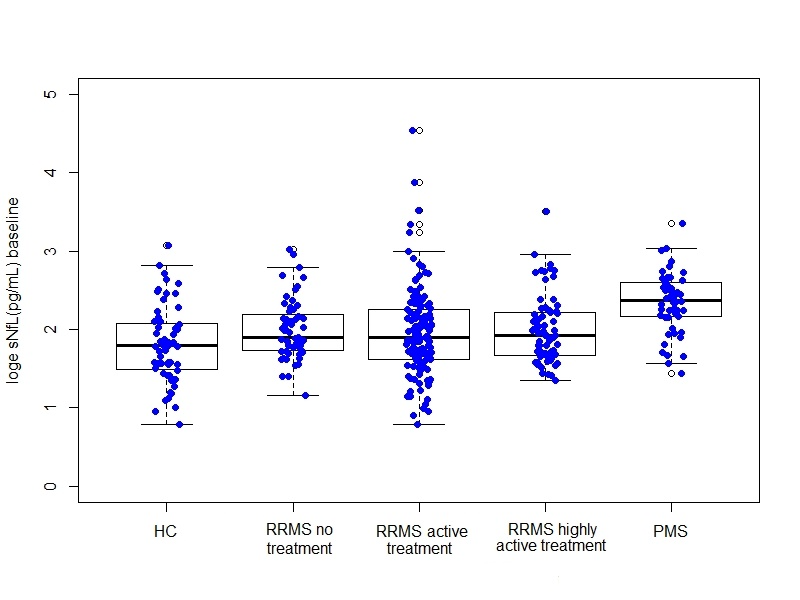

Supplement: sj-tiff-5-msj-10.1177_13524585221097296 – Supplemental material for Serum neurofilament light chain concentration predicts disease worsening in multiple sclerosis [file sj-tiff-5-msj-10.1177_13524585221097296.tiff]

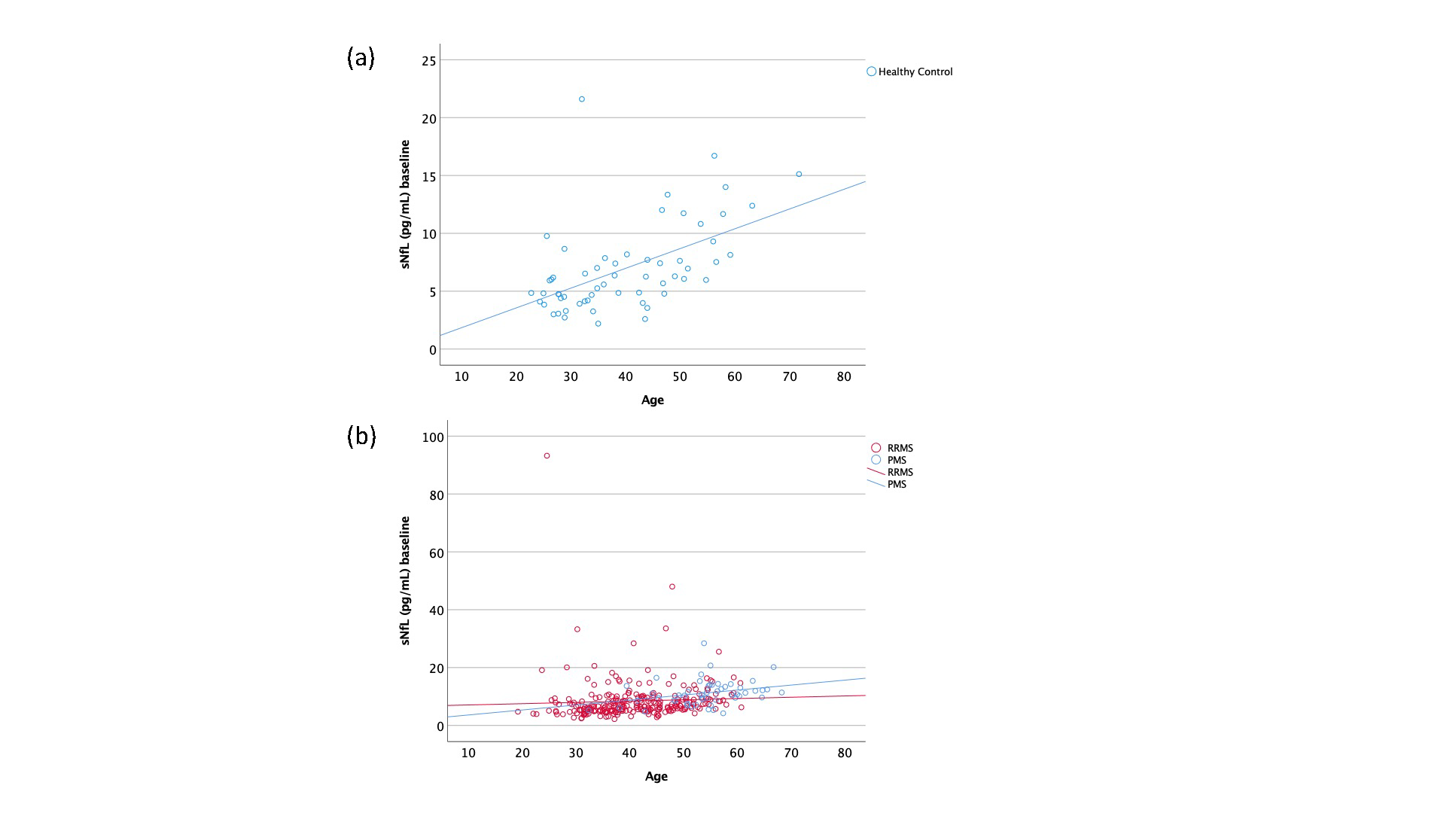

Supplement: sj-tiff-6-msj-10.1177_13524585221097296 – Supplemental material for Serum neurofilament light chain concentration predicts disease worsening in multiple sclerosis [file sj-tiff-6-msj-10.1177_13524585221097296.tiff]

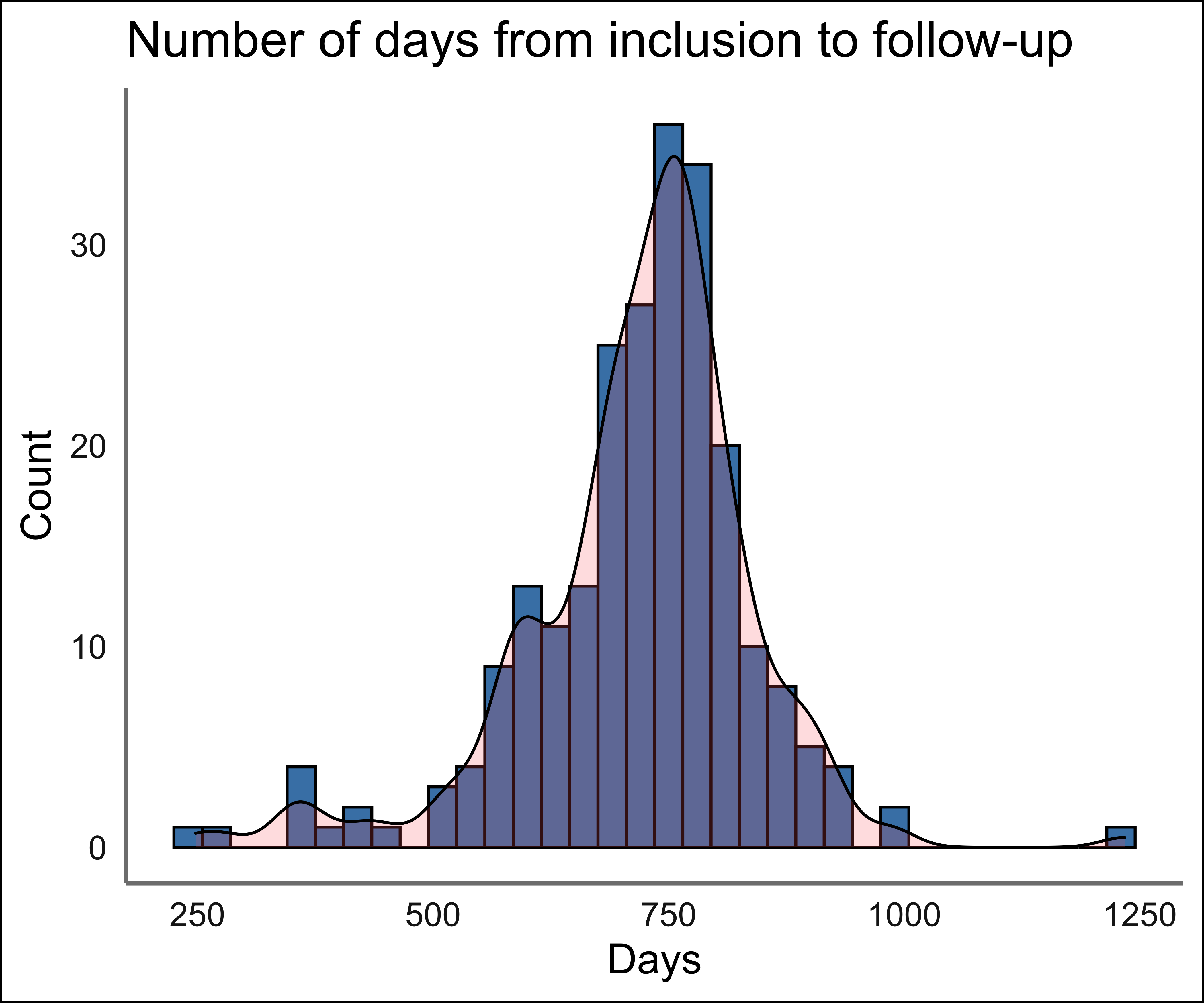

Supplement: sj-tiff-7-msj-10.1177_13524585221097296 – Supplemental material for Serum neurofilament light chain concentration predicts disease worsening in multiple sclerosis [file sj-tiff-7-msj-10.1177_13524585221097296.tiff]
